# Epstein-Barr virus infection promotes T cell dysregulation in a humanized mouse model of multiple sclerosis

**DOI:** 10.1126/sciadv.adu5110

**Published:** 2025-03-05

**Authors:** Jessica R. Allanach, Naomi M. Fettig, Blair K. Hardman, Ariel R. Rosen, Vina Fan, Cynthia Chung, Erin J. Goldberg, Zachary J. Morse, Iryna Shanina, Galina Vorobeychik, Lisa C. Osborne, Marc S. Horwitz

**Affiliations:** ^1^ Department of Microbiology and Immunology, The University of British Columbia, Vancouver, BC, Canada.; ^2^ Fraser Health Multiple Sclerosis Clinic, Burnaby, BC, Canada.; ^3^ Division of Neurology, Department of Medicine, University of British Columbia, Vancouver, BC, Canada.

## Abstract

Latent infection with Epstein-Barr virus (EBV) is a strong risk factor for the development of multiple sclerosis (MS), although the underlying mechanisms remain unclear. To investigate this association, we induced experimental autoimmune encephalomyelitis (EAE) in immunodeficient mice reconstituted with peripheral blood mononuclear cells (PBMCs) from individuals with or without a history of EBV infection and/or relapsing MS (RRMS). HuPBMC EAE mice generated from EBV-seronegative healthy donors were less susceptible to developing severe neurological symptoms than healthy EBV-seropositive and RRMS donor groups. Donor EBV seropositivity and RRMS diagnosis were associated with a significant increase in the number of central nervous system (CNS) infiltrating effector T cells due to enhanced proliferation of proinflammatory T cells and limited expansion of regulatory T cells. The data indicate that a history of EBV infection, further compounded by a diagnosis of RRMS, promotes T cell–mediated xenogeneic CNS disease in a humanized mouse model of MS.

## Teaser

Humanized mice derived from Epstein-Barr virus–infected donors exhibit worsened CNS disease due to dysregulated T cell response.

## INTRODUCTION

Multiple sclerosis (MS) is an autoinflammatory disease of the central nervous system (CNS) characterized by lesions of demyelinated nerve axons, infiltration of autoreactive T cells, and ongoing neurodegeneration (*1*). Clinical presentation among affected individuals is heterogeneous, with variable symptoms and disease courses ranging from relapsing-remitting (RRMS) to progressive forms, with intermediate phenotypes (*2*). Symptoms also vary widely but typically involve some combination of loss of coordination and sensation, muscle weakness, vision impairment, fatigue, pain, and cognitive impairment, among others (*3*). MS is especially prevalent among women, for whom there is a heavy bias in incidence (*4*). Although the exact etiology of MS is unclear, development of the disease is thought to be a consequence of environmental triggers in genetically susceptible individuals (*5*).

The environmental factor most strongly and consistently linked to MS is infection with Epstein-Barr virus (EBV) (*6*–*12*). EBV is a human gammaherpesvirus transmitted through saliva that infects more than 90% of all adults (*13*). During acute infection, the virus infects epithelial cells in the oropharynx and, subsequently, disseminates and infects memory B cells, within which the virus establishes latency and persists for the life of the host (*13*, *14*). EBV reactivates intermittently to transmit to new cells and hosts but is typically quiescent and, in healthy individuals, does not cause disease. EBV infection is, however, associated with a broad spectrum of malignancies and diseases (*13*, *15*). The varied pathological consequences of EBV infection are considered to be because of host-specific differences in immune responses to infection dictated by age, genetic predispositions, and confounding environmental exposures (*13*, *16*–*19*). Acute infection is usually asymptomatic when the virus is acquired at an early age, but delayed acquisition can cause infectious mononucleosis (IM): a self-limiting clinical condition marked by a large expansion of CD8^+^ T cells specific to viral antigens (*20*). The connection between EBV infection and MS was first posited because of the prominent epidemiological overlap in cases of IM and MS (*21*). Numerous studies have since provided evidence linking EBV infection with the development of MS (*6*, *7*, *9*–*12*).

Individuals who are EBV-seronegative (EBV^−^) have a relatively low risk for MS, whereas asymptomatically infected individuals have a moderate risk, and those who have previously developed IM have a significantly higher risk for MS than those who acquired the virus asymptomatically (*7*, *8*). Additionally, nearly all adult and pediatric patients with MS are EBV seropositive (EBV^+^) (*22*–*24*), and patients with MS show higher EBV-specific antibody titers than age-matched controls in the years preceding disease onset (*6*). Patients with MS also show increased frequencies of EBV-specific T cells in the blood compared to unaffected individuals with a similar viral load, some of which are cross-reactive to myelin epitopes (*11*, *25*, *26*). The strength of the association between EBV infection and MS is highlighted by the relatively lower contribution of other known environmental factors to disease risk. The odds ratio for EBV infection in MS has been reported to be ~3.6, while the next two strongest risk factors, smoking and low vitamin D levels, are only ~1.6 and 1.4, respectively (*5*).

Recently, a longitudinal case-matched study demonstrated a 32-fold increased risk for MS and significant up-regulation of a biomarker of axonal degeneration in individuals that go on to develop MS specifically following EBV seroconversion in young adulthood (*10*). The authors noted that the strength of the association likely could not be matched by any other known or suspected risk factor for disease (*10*). The efficacy of B cell depletion therapies in patients with MS further suggests a role for EBV infection in disease, as anti-CD20 antibodies may target the viral reservoir (*27*). Moreover, EBV infection is associated with the development of other autoimmune diseases, including rheumatoid arthritis and systemic lupus erythematosus (*28*, *29*), which suggests a common mechanism by which EBV predisposes for autoimmunity. Latent gammaherpesvirus infection is known to modulate immune responses relevant to autoimmunity, including down-regulation of B cell apoptosis, suppression of regulatory T cell (T_reg_) function, and production of an interferon (IFN) I response (*30*–*32*). Despite mounting epidemiological and clinical evidence suggesting that EBV infection is a causative factor in MS, experimental investigation of underlying mechanisms has been limited because of the high prevalence of EBV in the general population, the preponderance of asymptomatic EBV infection years before MS onset that obscures analysis of early autoimmune processes where latent EBV infection and/or infected B cells may be acting to incite disease, and, because EBV only naturally infects humans, a lack of suitable animal models.

Due to the narrow tropism of latent EBV for human B cells and resulting inability to infect rodent immune cells, the animal models that have helped shaped our understanding of MS pathogenesis are not amenable to EBV infection directly. To address this species limitation, our group previously developed a mouse model wherein wild-type C57Bl/6 mice are latently infected with the rodent homolog of EBV, gammaherpesvirus 68 (γHV68), and then induced with experimental autoimmune encephalomyelitis (EAE), a widely used autoimmune model of MS (*6*, *10*, *33*–*36*). Mice latently infected with γHV68 before EAE induction experience a severe pathology more alike MS than uninfected EAE mice (*35*, *36*). The enhanced MS-like disease is characterized by greater clinical scores with earlier symptom onset and CNS infiltration of activated macrophages/microglia, cytotoxic CD8^+^ T cells, and T helper 1 (T_H_1)–skewed CD4^+^ T cells, leading to demyelinated lesions in the brain, as well as decreased CD4^+^ T_reg_ frequencies (*35*). Moreover, these effects were found to be exclusive to latent γHV68 infection, as acute infection with latency-deficient γHV68 and chronic infection with other persistent murine viruses was not able to enhance EAE (*35*). The immunopathological features of the γHV68-EAE model are hallmark characteristics of MS that are either far less pronounced or absent in classical EAE models (*37*, *38*), which strongly indicates that EBV might contribute to similar aspects of the pathogenesis of MS in humans. Although this modeling approach has been informative, a more complete understanding of how EBV modifies MS susceptibility could be gained from improved models of host-viral interactions.

Here, we sought to study the role of EBV latency in MS using humanized mice that contain reconstituted human immune systems naturally exposed to EBV. EBV infection of humanized mice has recapitulated various aspects of the viral immune response and pathogenesis of EBV-associated diseases observed in humans, including lytic and latent infections (*39*–*41*), lymphoproliferation and tumor formation (*42*–*44*), among others (*45*–*47*). Studies of EBV infection in humanized mice, however, have not reported CNS-localized inflammation leading to clinically overt motor deficits, as seen in classical EAE models of MS. To capture the effects of long-term latent EBV infection on immune function and neuroinflammation when challenged later in life, we induced EAE in immunodeficient mice reconstituted with peripheral blood mononuclear cells (PBMCs) isolated from healthy EBV^+^ or EBV^−^ adults. Because the vast majority of people living with MS are EBV^+^ (*24*, *48*), we also tested PBMCs from this donor group for neuroinflammatory potential. Supporting a role for EBV-mediated immunomodulation in MS susceptibility, we found that donor EBV serostatus clearly defined the time of EAE symptom onset and severity of clinical disease. Further, engraftment of PBMCs from EBV^+^ donors resulted in diminished T_reg_ expansion and enhanced effector T cell proliferation and infiltration of the CNS, suggesting that latent EBV infection may promote autoimmunity via dysregulation of T cell responses.

## RESULTS

### EBV^+^ and RRMS donor–derived PBMCs exacerbate clinical disease severity in HuPBMC EAE mice

**Fig. 1.**
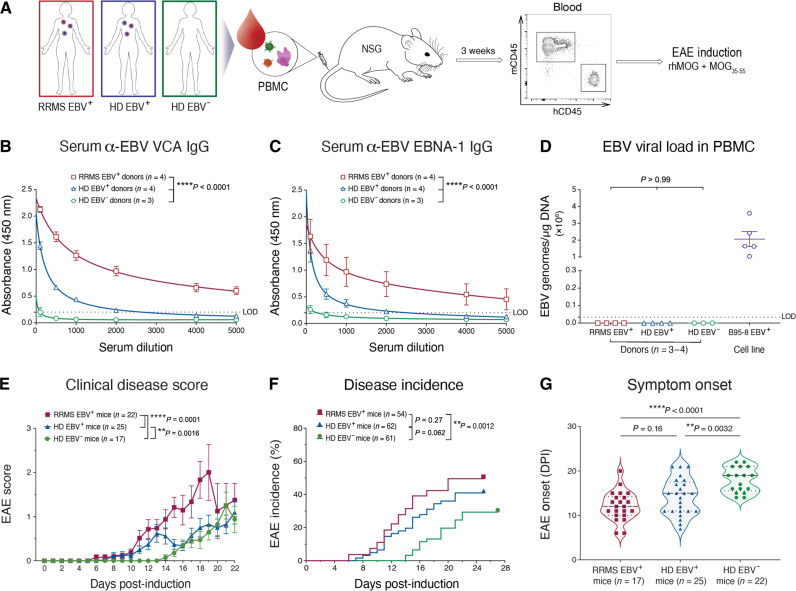
Donor EBV infection and RRMS diagnosis worsen clinical outcomes in HuPBMC EAE mice. (**A**) Experimental design: Donor PBMCs isolated from women with or without a history of EBV infection or an RRMS diagnosis were used to engraft immunocompromised NSG mice. Following a 3-week reconstitution period and confirmation of circulating human CD45^+^ cell repopulation, humanized NSG mice (HuPBMC) were immunized with recombinant human myelin oligodendrocyte glycoprotein (rhMOG) antigens to induce EAE. (**B**) Donor serum IgG specific to acute EBV antigen viral capsid antigen (VCA). (**C**) Donor serum immunoglobulin G (IgG) specific to latent EBV antigen Epstein-Barr nuclear antigen 1 (EBNA-1). In (B) and (C), group data are shown as means with SEM and were curve fit with a one-site total binding equation. Statistical differences in titer curves were assessed by ordinary two-way analysis of variance (ANOVA). (**D**) Cell-associated EBV viral loads in donor PBMCs measured by *BALF5* quantitative polymerase chain reaction (qPCR) assay. Data are shown as means with SEM and were analyzed by ordinary one-way ANOVA with Tukey’s multiple comparisons test. In (B) to (D), *n* = 3 to 4 donors per group and the lower limit of detection (LOD) for each assay is represented by a dotted line. (**E**) Clinical disease scores post-induction for symptomatic HuPBMC EAE mice. Data are shown as means with SEM, and curves were analyzed by ordinary two-way ANOVA (*n* = 17 to 25 mice per group derived from three to four donors per group). (**F**) Incidence of clinical EAE symptoms post-induction. Data are shown as percentage of the group, and curves were analyzed by log-rank (Mantel-Cox) test (*n* = 54 to 62 mice per group derived from three to four donors per group). (**G**) Day of EAE symptom onset post-induction (DPI). Distribution of individual data is shown with median and quartiles (dashed lines) and was analyzed by Brown-Forsythe and Welch ANOVA with Dunnett’s T3 multiple comparisons test (*n* = 17 to 25 mice per group derived from three to four donors per group).

To evaluate the impact of EBV infection on clinical outcomes of MS-associated disease, we engrafted NOD-*scid*-gamma (NSG) mice with PBMCs (HuPBMC mice) from three donor groups on the basis of EBV seropositivity and RRMS diagnosis, namely, EBV^+^ RRMS donors, EBV^+^ healthy donors (EBV^+^ HDs), and EBV^−^ healthy donors (EBV^−^ HDs) (Fig. 1A). For this study, we enrolled treatment-naïve female donors aged 19 to 39 because of the increased prevalence of RRMS among young women (*4*), who were all in clinical remission at the time of donation. Consistent with previous reports (*6*, *10*, *49*), the donors diagnosed with RRMS were all EBV^+^ and exhibited significantly elevated EBV-specific serum immunoglobulin G (IgG) titers to both the acute phase viral capsid antigen (VCA; Fig. 1B) and the latent-phase Epstein-Barr nuclear antigen 1 (EBNA-1; Fig. 1C) compared to previously infected, otherwise EBV^+^ HDs. Uninfected EBV^−^ HDs were identified and grouped on the basis of the absence of serum IgM and IgG specific to both EBV antigens. Evaluation of EBV^−^ RRMS donors was determined to be infeasible for this initial study because of the near universal EBV seropositivity among adults with MS (*10*, *48*). EBV viral load analysis of donor PBMCs confirmed that none of the donors had detectable levels of cell-associated EBV that might be indicative of a highly active infection or an EBV-associated disorder such as malignancy or IM (Fig. 1D) (*50*). We also assessed donor group differences in other serological factors associated with MS (*5*, *9*). Our donors showed 33 to 50% seropositivity for cytomegalovirus (CMV) within each donor group, which reflects the general population (Table 1) (*51*, *52*). Although there was no significant difference in anti-CMV IgG titers between EBV^+^ HD and the other two groups, EBV^+^ RRMS donors had elevated anti-CMV IgG compared to EBV^−^ HD (fig. S1A). We also verified that the donors did not have any existing overt seroreactivity to the inducing antigen, recombinant human myelin oligodendrocyte glycoprotein (rhMOG; fig. S1B), and that the donor groups had statistically similar serum 25-hydroxy vitamin D levels (fig. S1C). Human leukocyte antigen (HLA) typing revealed that two of the four RRMS donors expressed the MS risk allele DRB1*15:01 (*53*), whereas none of the HDs expressed this allele (fig. S1D). Individual donor characteristics, including clinical MS parameters and viral serostatus, are reported in Table 1.

**Table 1.** PBMC donor demographics, disease characteristics, and serology. All RRMS and healthy donors were female. RRMS participants were treatment naïve and in clinical remission at the time of donation. HD, healthy donor; EDSS, expanded disability status scale; VCA, viral capsid antigen; EBNA-1, Epstein-Barr nuclear antigen 1; CMV, cytomegalovirus; NA, not applicable.

Donor ID	Age (years)	Age (years) at MS symptom onset	EDSS score	Disease duration	EBV IgG serostatus	CMV IgG serostatus	Serum 25-OH (ng/ml)
MS-01	24	24	2.5	4 months	VCA +	+	29.7
					EBNA-1 +		
MS-02	31	31	2.5	6 months	VCA +	+	34.9
					EBNA-1 −		
MS-03	32	26	2.0	5 years	VCA +	−	19.9
					EBNA-1 +		
MS-04	39	31	2.0	7 years	VCA +	−	66.5
					EBNA-1 +		
HD-01	39	NA	NA	NA	VCA +	−	27.7
					EBNA-1 +		
HD-02	25	NA	NA	NA	VCA −	−	35.0
					EBNA-1 −		
HD-03	26	NA	NA	NA	VCA −	−	29.8
					EBNA-1 −		
HD-04	23	NA	NA	NA	VCA +	+	37.5
					EBNA-1 +		
HD-05	19	NA	NA	NA	VCA −	+	18.9
					EBNA-1 −		
HD-06	25	NA	NA	NA	VCA +	+	38.4
					EBNA-1 −		
HD-07	28	NA	NA	NA	VCA +	−	27.5
					EBNA-1 +		

Flow cytometric analysis of transplanted PBMCs confirmed that recipient NSG mice were engrafted with a similar composition of immune cells from each donor (fig. S2). HuPBMC mice were then induced with EAE using a mixture of rhMOG_1-120_ protein and MOG_35-55_ peptide after a 3-week engraftment period post–PBMC injection (Fig. 1A). HuPBMC mice were found to be susceptible to EAE and exhibited symptoms typical of other murine EAE models, including weight loss, piloerection, motor imbalance, and an ascending, monophasic paralysis, reaching EAE scores of up to 4 on a standard 5-point scale (*33*), with resolution within approximately a week from onset (figs. S3 and S9). The development of paralysis in this model appeared similar to the asynchronous, relapsing EAE phenotype of the nonobese diabetic (NOD) background strain for the NSG mouse (fig. S3) (*54*). HuPBMC EAE mice demonstrated an exacerbation of clinical disease by multiple measures when engrafted with PBMCs from donors who were EBV^+^ and had been diagnosed with RRMS (Fig. 1, E to G). The overall disease course of EAE was significantly worsened in EBV^+^ HD mice compared to that in EBV^−^ HD mice, which was, in turn, significantly more severe in the EBV^+^ RRMS recipient cohorts compared to that in both HD groups (Fig. 1E and fig. S4). The incidence of EAE symptoms was significantly greater in EBV^+^ RRMS recipient mice compared to that in the HD groups and moderately increased with EBV infection between HD groups (Fig. 1F). HuPBMC EAE mice derived from EBV^−^ HD donors also exhibited a significantly delayed time to symptom onset compared to those from the EBV^+^ recipient groups, which was not significantly different between the EBV^+^ recipient groups, regardless of an RRMS diagnosis (Fig. 1G). The clinical outcomes of EAE indicate that donor EBV infection and RRMS diagnosis both increase disease susceptibility and severity in recipient HuPBMC EAE mice.

### Human T cells infiltrate and localize to areas of inflammatory demyelination in the CNS of HuPBMC EAE mice

**Fig. 2.**
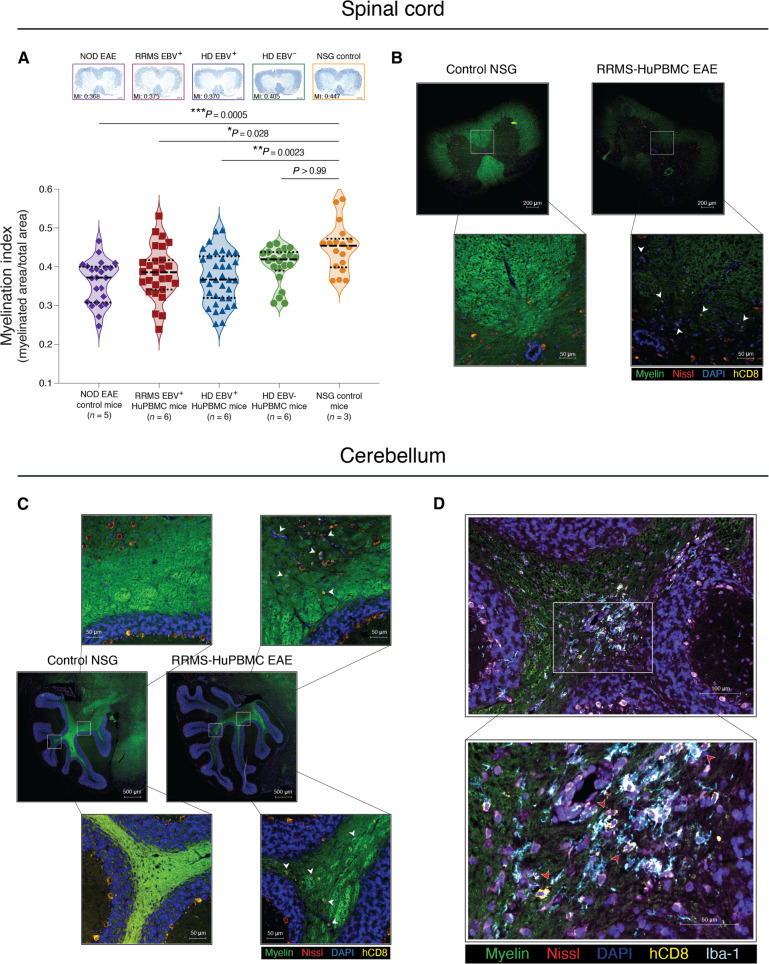
EAE induction in HuPBMC mice causes human T cell infiltration and inflammatory demyelination of the CNS. (**A**) Demyelination of the spinal cord in the HuPBMC EAE model. Perfused spinal cords were obtained days 19 to 25 post-induction (5 to 8 days post–symptom onset) from HuPBMC EAE mice and days 15 to 25 post-induction from NOD EAE mice (5 to 15 days post–symptom onset). Eriochrome cyanine–stained sections (top) obtained from the lower thoracic region of the spinal cord show representative myelination indices (MI) for each of the respective group means. Individual data points represent averages of serial sections sampled from four to six equidistant regions along the entire length of the spinal cord (*n* = 18 regional points from three unengrafted NSG control mice; *n* = 22 to 36 regional points from five to six mice per group for EAE-induced NOD and HuPBMC groups). Distribution of individual data is shown with median and quartiles (dashed lines) and was analyzed by Kruskal-Wallis with Dunn’s multiple comparisons test. (**B** to **D**) Human CD8^+^ cells infiltrate the CNS of HuPBMC EAE mice engrafted with RRMS donor PBMCs. Representative images of lumbar spinal cord (B) and cerebellar (C and D) sections from an unengrafted control NSG mouse (left) and a symptomatic HuPBMC EAE mouse (right) derived from a donor with RRMS. Perfused tissues were collected day 15 post–EAE induction (day 4 post–symptom onset). Sections were labeled with FluoroMyelin (green), NeuroTrace 530/615 (red), 4′,6-diamidino-2-phenylindole (DAPI; blue), anti-hCD8 (yellow), and anti–Iba-1 (light blue). Example hCD8^+^ cells are indicated by white arrowheads (B and C), and hCD8^+^ cells in proximity to Iba-1^+^ cells by red arrowheads (D). Scale bars indicate size as specified per panel, showing (A) 200 μm; (B) 200 μm and (insets) 50 μm; (C) 500 μm and (insets) 50 μm; and (D) 100 μm and (inset) 50 μm.

In classical EAE models, clinical disease scores are assessed on the basis of the severity of ascending paralysis, which is a consequence of inflammatory damage to the spinal cord (*37*). To investigate the underlying cause of the clinical symptom differences associated with donor EBV and RRMS status, we first evaluated the degree of demyelination of the spinal cord among the three recipient HuPBMC EAE groups. EAE induction of immunocompetent NOD mice and RRMS/HD EBV^+^ HuPBMC mice led to a significant loss of total myelin content relative to unaffected NSG control mice (Fig. 2A). Both EBV^+^ groups had significantly less myelin in the spinal cord compared to NSG controls, whereas EAE induction of EBV^−^ HD recipient HuPBMC mice did not significantly reduce myelination, indicating greater protection from immune cell–mediated EAE demyelination consistent with diminished clinical disease severity in this group. As demyelination in EAE is predominantly T cell driven (*55*, *56*), we confirmed that engrafted human T cells infiltrated the CNS following induction. Notably, we detected an accumulation of human CD8^+^ T cells in the white matter of both the spinal cord (Fig. 2B) and the cerebellum (Fig. 2C) of HuPBMC EAE mice engrafted with RRMS donor PBMCs, demonstrating that engrafted human T cells can localize to CNS regions typically demyelinated in classical EAE models. Moreover, we observed an inflammatory lesion within the cerebellum marked by microgliosis, as indicated by concentrated ionized calcium-binding adaptor molecule-1 (Iba-1) staining, that co-localized with human CD8^+^ T cell infiltration (Fig. 2D). Demyelination of the spinal cord and brain lesion infiltrates composed of macrophages and CD8^+^ T cells are consistent with the CNS immunopathology seen in both EAE models and MS, respectively (*38*, *57*).

### CNS immunopathology in HuPBMC EAE is mediated by human T_H_1 and cytotoxic T cells and murine macrophages

**Fig. 3.**
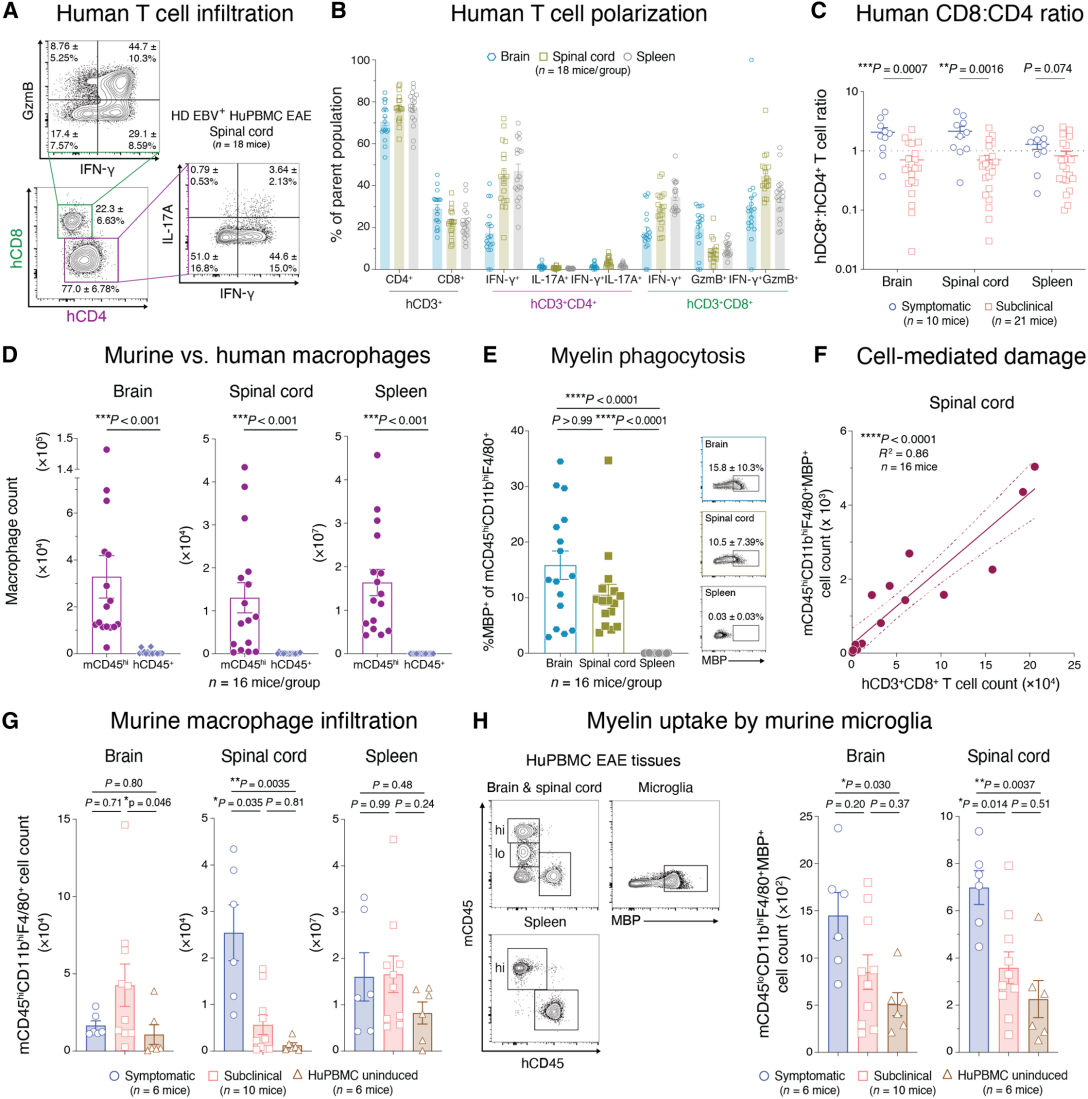
EAE in HuPBMC mice is mediated by infiltrating human T_H_1 and cytotoxic T cells and by murine myeloid cells. (**A**) Spinal cord–infiltrating inflammatory T cell subsets. (**B**) Human T cell subsets quantified in whole tissues. In (A) and (B), perfused tissues were collected day 22 post-induction, and cells were stimulated with phorbol 12-myristate 13-acetate (PMA) and ionomycin (*n* = 18 mice from one EBV^+^ HD). (**C**) Human CD8^+^:CD4^+^ T cell ratios in symptomatic or subclinical HuPBMC EAE tissues (*n* = 10 to 21 mice per group). (**D**) Numbers of murine (mCD45^hi^CD11b^hi^F4/80^+^) and human (hCD45^+^CD14^+^CD68^+^) macrophages in HuPBMC EAE tissues (*n* = 16 mice per group). (**E**) Frequency of murine macrophages containing intracellular myelin basic protein (MBP) in HuPBMC EAE tissues (*n* = 16 mice per group). (**F**) Correlation between numbers of spinal cord–infiltrating hCD3^+^CD8^+^ T cells and murine macrophages containing intracellular MBP (*n* = 16 mice). (**G**) Numbers of infiltrating murine macrophages in the tissues of symptomatic and subclinical HuPBMC EAE mice or uninduced HuPBMC control mice (*n* = 6 to 10 mice per group). (**H**) Numbers of murine microglia containing intracellular MBP in the CNS of symptomatic and subclinical HuPBMC EAE mice or uninduced HuPBMC control mice (*n* = 6 to 10 mice per group). In (C) to (H), perfused tissues were collected days 14 to 24 post-induction of cohorts derived from two to three unrelated EBV^+^ HDs, and data were combined for analysis. In [(B) to (D)], [(E), (G), and (H)], data are shown as means with SEM. In [(C) and (D)], data were analyzed by Mann-Whitney test. In [(E), (G), and (H)], data were analyzed by Kruskal-Wallis with Dunn’s multiple comparisons test or by Brown-Forsythe and Welch ANOVA with Dunnett’s T3 multiple comparisons test. In (F), data were analyzed by simple linear regression (dashed lines show 95% confidence interval). Concatenated flow plots show mean frequency of parent population ± SD.

Murine EAE models can present with a range of distinct disease symptomologies, CNS pathologies, and constituent autoimmune processes based on the induction method and rodent strain used. We first characterized the immunopathological features of EAE mice using NSG mice engrafted with HD EBV^+^ PBMCs to establish a baseline from which to compare EBV^−^ and RRMS PBMC recipient cohorts. Consistent with our findings by histology, we measured substantial spinal cord infiltration of both human CD4^+^ (hCD4^+^; 3802 ± 3525 cells per spinal cord) and CD8^+^ (hCD8^+^; 1225 ± 1262 cells per spinal cord) T cells by flow cytometry in HuPBMC EAE mice (Fig. 3A). A large proportion of hCD4^+^ T cells in the CNS produced IFN-γ (~20 to 45%). Although there were relatively few interleukin-17A (IL-17A)^+^ cells (~1 to 4%), the majority of these cells co-expressed IFN-γ, suggesting that this model is predominantly IFN-γ^+^ T_H_1 driven (Fig. 3, A and B). Among CNS-infiltrating hCD8^+^ T cells, ~80% expressed IFN-γ and/or granzyme B (GzmB) (Fig. 3, A and B), indicating that they are capable of cytotoxic activity. Although the level of T cell reconstitution and infiltration was variable among tissues derived from the same recipient cohort, the pattern of cytokine expression was similar in the periphery and in the CNS (Fig. 3B). Human T cell infiltration was detected in the CNS of all EAE-induced HuPBMC mice regardless of the development of clinically visible EAE symptoms (fig. S5). However, the spinal cords of mice that developed clinical symptoms contained significantly greater numbers of hCD45^+^ cells, including total hCD3^+^ and activated hCD8^+^ T cells, than those of mice that remained subclinical (fig. S5). Moreover, there was a significantly higher ratio of hCD8^+^ T cells relative to hCD4^+^ T cells within the CNS of mice with EAE symptoms (Fig. 3C), suggesting a critical role for cytotoxic hCD8^+^ T cells in mediating xenogeneic demyelination and tissue damage.

Myelin phagocytosis by infiltrating and resident macrophages/microglia is a key mediator of demyelination in EAE models (*58*, *59*). In humanized mouse models, both endogenous murine and engrafted human myeloid cells reconstitute and can coexist within the CNS (*60*, *61*). We assessed the abundance of murine (mCD45^hi^CD11b^hi^F4/80^+^) and human (hCD45^+^CD14^+^CD68^+^) macrophages in the CNS and periphery to determine the potential relative contribution of each subset to the immunopathology of HuPBMC EAE. Unlike hematopoietic stem cell engraftment–based humanized mouse models, PBMC humanization of NSG mice favors the engraftment of T cells over innate immune subsets (*62*). Accordingly, we observed numerous mCD45^hi^ macrophages in the brains, spinal cords, and spleens of HuPBMC EAE mice but very few hCD45^+^ macrophages (Fig. 3D). The overall scarcity of hCD45^+^CD14^+^CD68^+^ cells in the CNS suggests that infiltrating murine macrophages and resident microglia likely perform the bulk of axonal demyelination following EAE induction. We confirmed that murine myeloid cells phagocytosed myelin in the HuPBMC EAE model on the basis of the detection of intracellular myelin basic protein (MBP) in mCD45^hi^ macrophages in the CNS after immunization with MOG antigen (Fig. 3E). The total number of CNS-infiltrating hCD8^+^ T cells correlated strongly and specifically with the number of infiltrating MBP^+^ murine macrophages in the same tissue (Fig. 3F and fig. S6), similar to MS brain lesions wherein infiltrating cytotoxic T cells and macrophages are positively correlated with the extent of axonal damage (*63*). hCD8^+^ T cell and murine macrophage counts correlated significantly in the brain and even more so in the spinal cord (Fig. 3F and fig. S6, A and B), the dominant site of demyelination in EAE models (*37*). An association with myelin phagocytosis was not observed for hCD4^+^ T cells or human macrophages in either tissue (fig. S6, C to F). Furthermore, we observed a significant increase in the total numbers of murine macrophages and myelin-phagocytosing microglia (mCD45^lo^CD11b^hi^F4/80^+^MBP^+^) in the spinal cords of HuPBMC mice that developed clinical EAE symptoms compared to those of mice that remained subclinical (Fig. 3, G and H), supporting the notion that murine myeloid cells demyelinate the CNS sufficiently to produce clinical symptoms in this model.

Collectively, the data indicate that infiltrating hCD4^+^ T cells direct a T_H_1-polarized response in the CNS of HuPBMC EAE mice that leads to hCD8^+^ cytotoxic T cell–mediated myelin damage and subsequent phagocytosis by murine macrophages and microglia. Immunization of HuPBMC mice with MOG antigen emulsified in Freund’s complete adjuvant (FCA), as compared to that in blank FCA, resulted in a 30% increase in the incidence of symptomatic disease, indicating that the inclusion of myelin antigen immunization preferentially directed the xenogeneic graft response of the engrafted human T cells to the mouse CNS (fig. S3, E and F). These data highlight the interspecies nature of the immunopathology of the HuPBMC EAE model and support its use as a model of MS.

### Donor EBV seropositivity and RRMS diagnosis promote effector T cell accumulation in the CNS of HuPBMC EAE mice

**Fig. 4.**
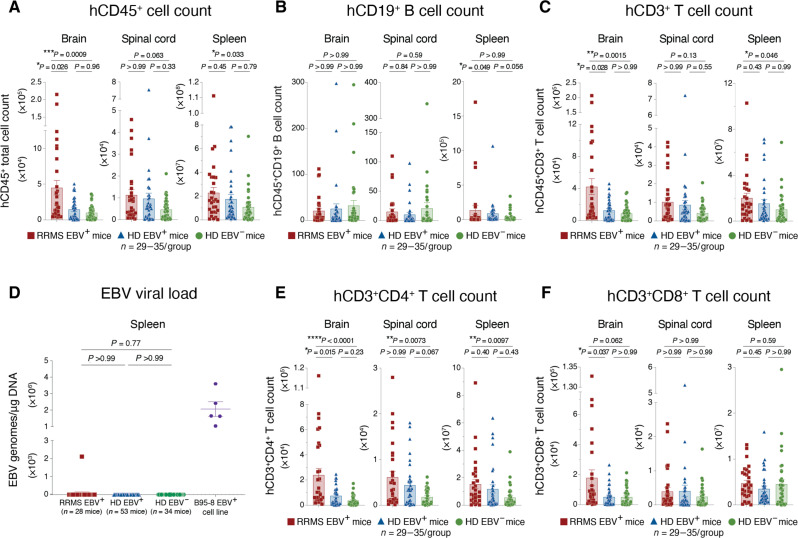
Donor EBV and RRMS status promote human immune cell infiltration of the HuPBMC EAE CNS without viral reactivation. (**A**) Total human CD45^+^ immune cell counts, (**B**) hCD19^+^ B cell counts, (**C**) hCD3^+^ T cell counts, (**D**) EBV genome copies in splenic DNA, (**E**) hCD3^+^CD4^+^ T cell counts, and (**F**) hCD3^+^CD8^+^ T cell counts in whole brains, spinal cords, and spleens of recipient HuPBMC EAE mice at endpoint, grouped by PBMC donor EBV serostatus and RRMS diagnosis. Perfused organs were collected days 14 to 27 post–EAE induction (average 5 to 10 days post–symptom onset). For total immune cell quantification, *n* = 29 to 35 mice per group derived from two to three donors per group. For viral load quantification, *n* = 28 to 53 mice per group derived from two to four blood donors per group; *n* = 5 replicates for control EBV^+^ B95-8 cell line, and assay lower limit of detection is represented by a dashed line. All data are shown as means with SEM and were analyzed by Kruskal-Wallis with Dunn’s multiple comparisons test.

Because the HuPBMC EAE recipient groups showed an EBV- and RRMS-associated increase in disease severity and demyelination, we sought to assess human immune cell infiltration of the CNS and reconstitution in the periphery to identify contributing immune subsets. In the brain, spinal cord, and spleen, HuPBMC EAE recipient cohorts followed a variable, stepwise trend of increased total human CD45^+^ immune cell counts with donor EBV seropositivity and RRMS diagnosis (Fig. 4A). In all HuPBMC EAE mice, relatively few human B cells infiltrated the CNS relative to T cells: ~25 to 100 hCD19^+^ B cells versus 10^3^ to 10^5^ hCD3^+^ T cells per spinal cord (Fig. 4, B and C). Total B cell infiltration of the CNS and reconstitution in the periphery did not differ between the three recipient groups (Fig. 4B).

EBV intermittently reactivates from latently infected memory B cells to transmit to new cells (*64*), which have been suggested to potentially contribute to relapses in MS (*65*). Although engrafted B cells reconstituted the spleen (~10^3^ to 10^5^ hCD19^+^ B cells), EBV viral genomes were undetectable in the spleens of most mice at EAE endpoint, regardless of recipient group (Fig. 4D), indicating that peripheral viral reactivation from engrafted B cells is unlikely to have influenced disease outcomes in this study. We further assessed whether an active EBV infection may play a role in promoting disease by measuring antibody production by EBV-specific B cells. Given that engrafted lymphocytes do not form organized germinal centers in NSG mouse lymphoid tissues and are subsequently unable to promote class switching and hypermutation of human B cells (*66*, *67*), any EBV-specific IgG produced in these mice would indicate a reactivation of engrafted memory B cells rather than a novel response to infection post-engraftment. We confirmed the production of rhMOG-specific human IgM and the absence of rhMOG-specific human IgG in the serum of HuPBMC EAE mice (fig. S7A), indicating the engraftment of otherwise functionally responsive human B cells. In three separate EBV^+^ recipient cohorts, no human IgG to the EBV antigen VCA could be detected in serum after EAE induction (fig. S7B), despite a strong response in corresponding donor serum (Fig. 1B), suggesting that, under these conditions, an active infection likely does not play a role in the enhancement of clinical disease.

Although direct B cell involvement in causing disease differences appeared improbable, total hCD3^+^ T cell counts comprised most of the infiltrating human CD45^+^ cells in the CNS, following the same increasing trend of higher counts with donor EBV and RRMS status (Fig. 4C). Among infiltrating T cells, hCD4^+^ T cells demonstrated a consistent increasing trend in total counts in all three tissues with donor EBV seropositivity and RRMS (Fig. 4E), while hCD8^+^ T cell counts were increased only in the brain of RRMS recipient mice compared to that of HD mice (Fig. 4F). Increased hCD8^+^ T cell counts in the RRMS recipient brains corresponded to greater numbers of infiltrating murine immune cell in these samples (fig. S7C), whereas microglia and human macrophage counts did not differ between recipient groups in any tissue (fig. S7, D and E). Splenic reconstitution of hCD8^+^ T cells was also similar between groups (Fig. 4F). Overall, EBV serostatus, separate from an RRMS diagnosis, most notably correlated with increased total numbers of hCD4^+^ T cells infiltrating the spinal cord (Fig. 4E), which aligns with increased protection from demyelination observed in EBV^−^ HD mice compared to that in EBV^+^ recipients (Fig. 2).

**Fig. 5.**
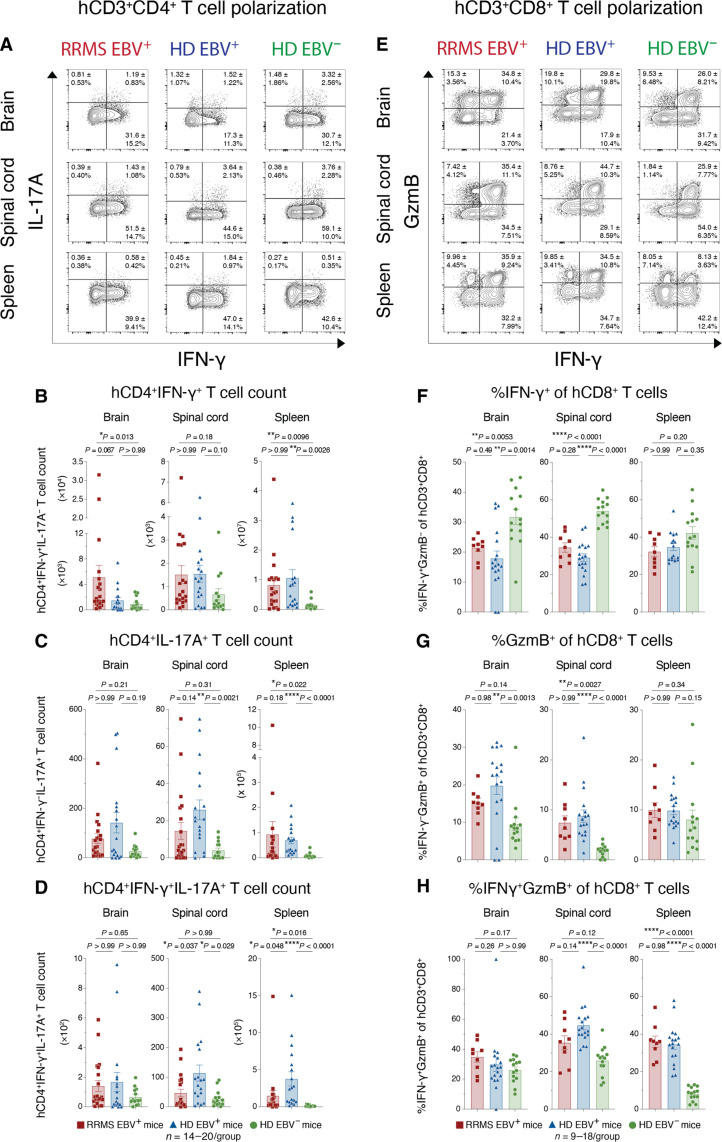
Donor EBV and RRMS status promote effector T cell expansion in the HuPBMC EAE model. Figure shows whole brain and spinal cord infiltration and spleen reconstitution in recipient HuPBMC EAE mice at endpoint, grouped by PBMC donor EBV serostatus and RRMS diagnosis. (**A**) Concatenated flow cytometric plots of IFN-γ and IL-17A expression, showing the mean frequency of hCD3^+^CD4^+^ cells ± SD, as well as corresponding total (**B**) IFN-γ^+^(IL-17A^−^), (**C**) IL-17A^+^(IFN-γ^−^), and (**D**) IFN-γ^+^IL-17A^+^ hCD4^+^ T cell counts in each tissue. (**E**) Concatenated flow cytometric plots of IFN-γ and GzmB expression, showing the mean frequency of hCD3^+^CD8^+^ cells ± SD, quantified as (**F**) %IFN-γ^+^(GzmB^−^), (**G**) %GzmB^+^(IFN-γ^−^), and (**H**) %IFN-γ^+^GzmB^+^ of hCD3^+^CD8^+^ cells in each tissue. Perfused organs were collected days 14 to 27 post–EAE induction (average 5 to 10 days post–symptom onset). Isolated immune cells were stimulated with PMA and ionomycin for cytokine detection (*n* = 9 to 20 mice per group derived from one to two donors per group). All plotted data are shown as means with SEM and were analyzed by Brown-Forsythe and Welch ANOVA with Dunnett’s T3 multiple comparisons test or by Kruskal-Wallis with Dunn’s multiple comparisons test.

Among hCD4^+^ T cells, the proportions of cells expressing cytokines indicative of subset skewing (T_H_1 and T_H_17) did not follow any consistent trends with PBMC donor EBV or RRMS status (Fig. 5A and fig. S8, A to C), indicating that polarization toward a particular inflammatory T_H_ subset was not the main driver of clinical group differences. However, the increased number of total hCD4^+^ T cells in EBV^+^ HD and RRMS recipient group tissues than those in EBV^−^ HD tissues (see Fig. 4E) led to an overall greater abundance of effector T_H_1 cells in the CNS and spleen (Fig. 5B), despite similar levels of IFN-γ expression (Fig. 5A and fig. S8A). The relatively fewer total numbers of T_H_17 (Fig. 5C) and IFN-γ^+^IL-17Α^+^ T_H_ cells (Fig. 5D) showed some moderate but not notable group-specific trends that would, otherwise, suggest that they underpinned the observed clinical disease differences. Despite similar numbers of total infiltrating hCD8^+^ T cells in the CNS between recipient cohorts (see Fig. 4F), donor EBV seropositivity led to significantly enhanced T cell cytotoxicity (Fig. 5E), as measured by a significant shift from less IFN-γ expression (Fig. 5F) to more GzmB expression (Fig. 5, G and H) among hCD8^+^ T cells in the brain and spinal cord. This trend was not further enhanced by donor RRMS diagnosis. Although total effector hCD8^+^ T cell counts were similar between recipient group tissues (fig. S8, D to F), the skewing toward increased cytotoxic capacity within the infiltrating hCD8^+^ T cell population is consistent with the elevated level of demyelination in the CNS of EBV^+^ recipient mice (see Fig. 2A). Overall, both a donor history of EBV infection and a diagnosis of RRMS led to incremental increases in the accumulation of pathogenic effector hCD4^+^ and hCD8^+^ T cells in the CNS of HuPBMC mice induced with EAE.

### Donor EBV seropositivity and RRMS diagnosis limit T_reg_ expansion in HuPBMC EAE mice

**Fig. 6.**
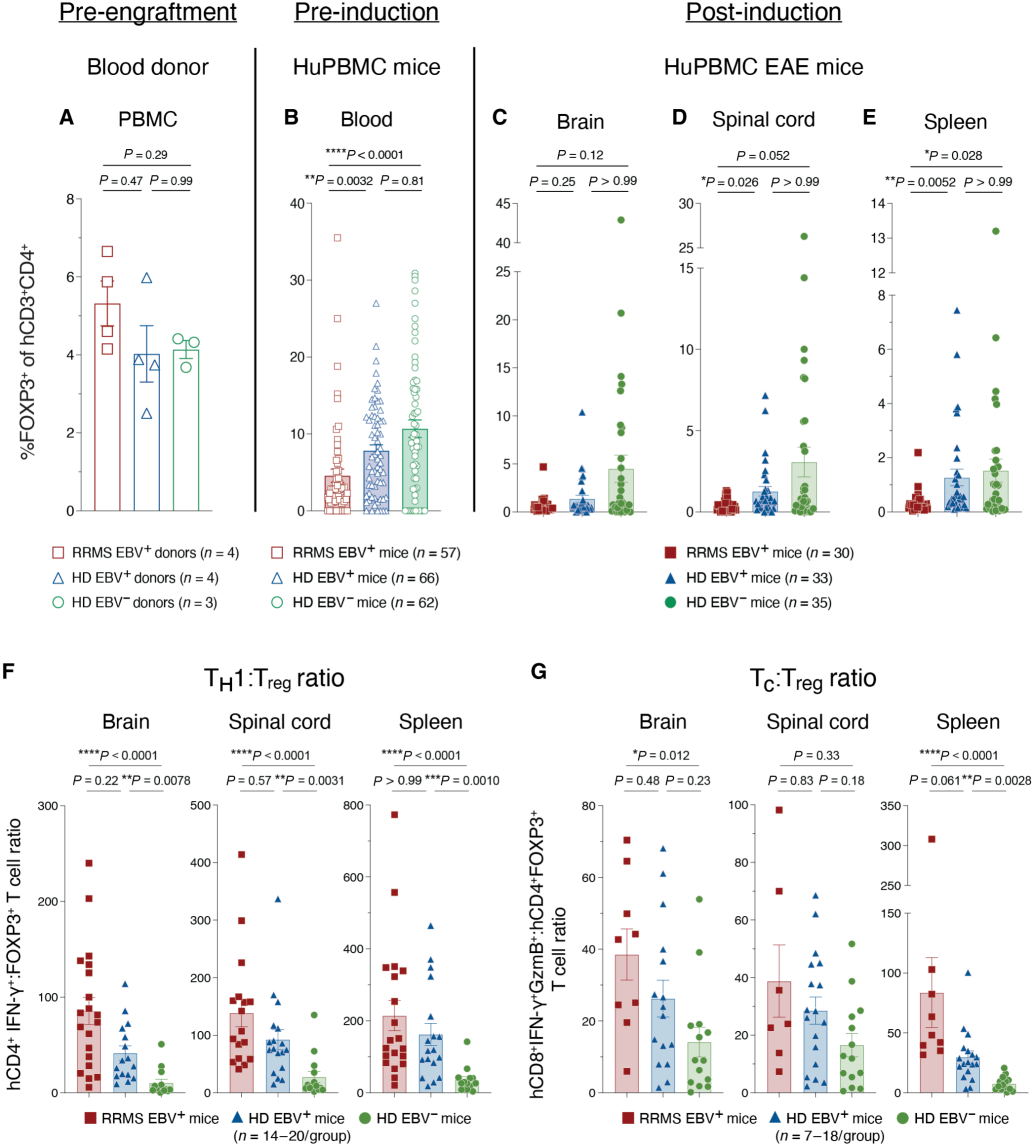
Donor EBV and RRMS status limit T_reg_ expansion in HuPBMC EAE mice. The proportion of hCD3^+^CD4^+^ T cells expressing FOXP3 in (**A**) freshly isolated donor PBMC (*n* = 3 to 4 donors per group), (**B**) in the peripheral blood of engrafted HuPBMC mice at 3 weeks post–PBMC injection (*n* = 57 to 62 mice per group derived from three to four donors per group), and in the (**C**) brain, (**D**) spinal cord, and (**E**) spleen of HuPBMC EAE mice at endpoint (*n* = 30 to 35 mice per group derived from two to three donors per group). The ratio of infiltrating (**F**) hCD4^+^IFN-γ^+^ (T_H_1) and (**G**) hCD8^+^IFN-γ^+^GzmB^+^ (T_c_) to regulatory hCD4^+^FOXP3^+^ (T_reg_) cells per tissue in recipient HuPBMC EAE mice at endpoint, grouped by PBMC donor EBV serostatus and RRMS diagnosis (*n* = 7 to 20 mice per group from one to two donors per group). Cells were isolated from perfused organs collected days 14 to 27 post–EAE induction (average 5 to 10 days post–symptom onset) and, for cytokine detection, stimulated with PMA and ionomycin. Data are shown as means with SEM and were analyzed by Brown-Forsythe and Welch ANOVA with Dunnett’s T3 multiple comparisons test (A) or by Kruskal-Wallis with Dunn’s multiple comparisons test [(B) to (G)].

Alongside greater T_H_1 and cytotoxic effector T cell infiltration of the CNS, we measured a reduced proportion of hCD4^+^ T cells expressing the regulatory transcription factor FOXP3 in RRMS recipient group tissues compared to those in the HD groups (Fig. 6). Initially, the FOXP3^+^ proportion of hCD4^+^ T cells from freshly isolated PBMCs was comparable between donor groups (Fig. 6A) but was significantly lower among RRMS hCD4^+^ T cells in the blood of NSG mice following engraftment of the PBMCs compared to those of HD engrafted mice (Fig. 6B). Following EAE induction, this trend of lower proportions of FOXP3^+^ T cells was, likewise, observed in the CNS and spleen of RRMS recipient mice (Fig. 6, C to E), although total hCD4^+^FOXP3^+^ T_reg_ counts were similar between groups at endpoint (fig. S7F). The sum consequence of this incremental increase in pathogenic T cell infiltration of the CNS and simultaneous lack of T_reg_ expansion was a significantly greater ratio of effector hCD4^+^ and hCD8^+^ T cells to regulatory hCD4^+^ T cells in the CNS and periphery of EBV^+^ HD and RRMS recipient mice compared to those in EBV^−^ HD mice (Fig. 6, F and G, and fig. S7, G and H).

As sample collection was timed to maintain similar overall cumulative EAE scores between groups at endpoint, differences in CNS infiltration could not be correlated with greater clinical disease burden for RRMS and EBV^+^ recipient groups (fig. S9, A to D). Although weight loss followed the EBV and RRMS status trend seen with clinical disease severity (fig. S9E), the incidence of clinical symptoms of xenogeneic graft-versus-host disease (xGvHD) was comparably low between the groups and did not follow the same trend with EBV and RRMS status (fig. S9, F to I). The data collectively suggest that a history of both EBV infection and an RRMS diagnosis among donors leads to a compounded reduction in the expansion of T_regs_ and a concomitant increase in effector T_H_1 and cytotoxic T cell abundance, and, subsequently, to systemic dysregulation of xenogeneic T cell–mediated inflammation, resulting in excessive CNS damage and worsened clinical disease outcomes following EAE induction.

### EBV infection and RRMS both enhance donor T cell proliferation following TCR stimulation

**Fig. 7.**
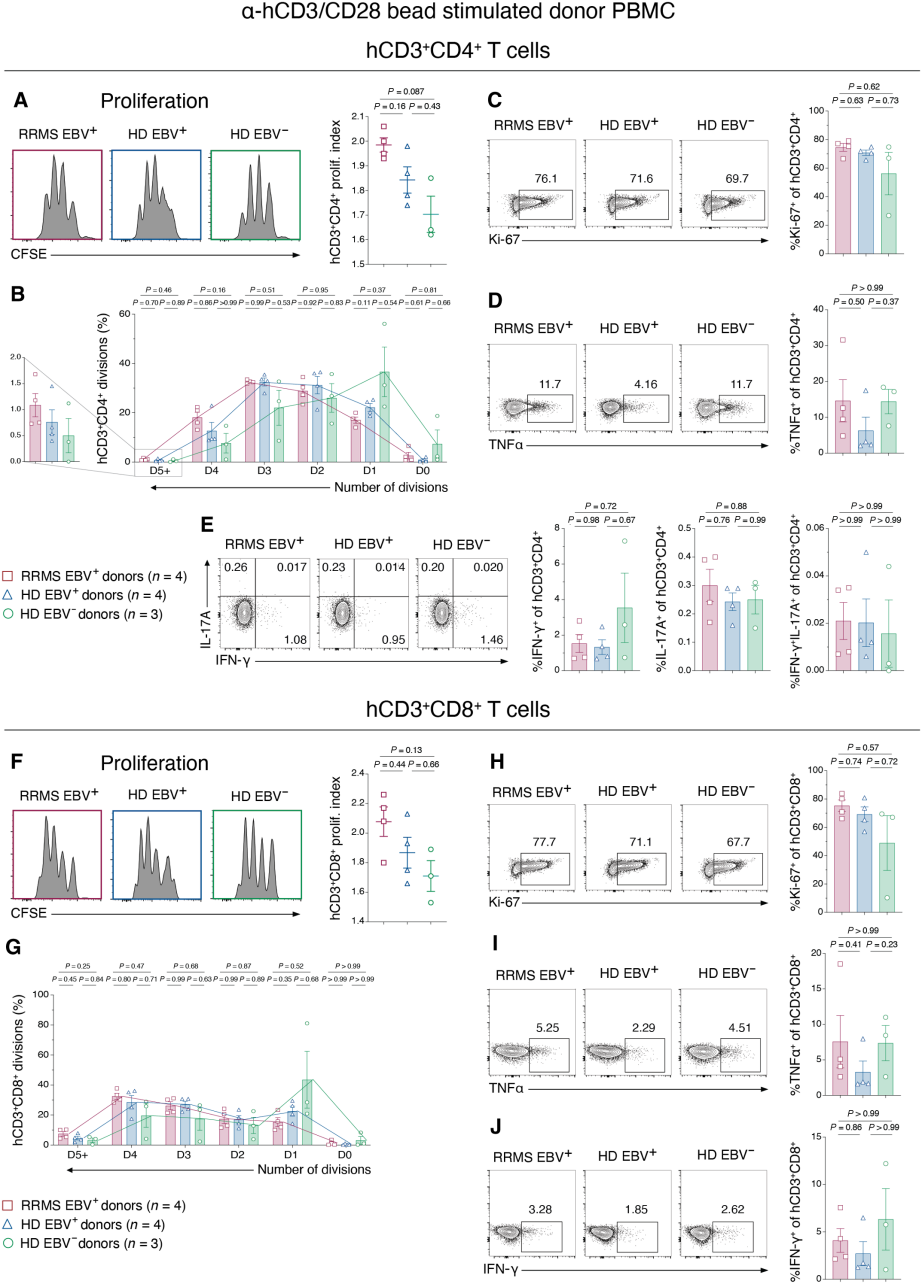
Donor T cell proliferation is enhanced by both EBV seropositivity and RRMS. Previously frozen, whole PBMC samples from EBV^+^ RRMS, EBV^+^ HD, and EBV^−^ HD blood donors were incubated with anti-CD3/CD28–coated beads for 96 hours to stimulate T cells in the absence of a specific antigen. Figure shows (**A**) the proliferation index determined by CFSE staining, (**B**) the proportion of hCD4^+^ T cells having undergone a specified number of cellular divisions by CFSE staining, (**C**) Ki-67 expression, (**D**) Tumor necrosis factor–α (TNFα) expression, and (**E**) IFN-γ and IL-17A expression on hCD3^+^CD4^+^ T cells, as well as (**F**) the proliferation index determined by CFSE staining, (**G**) the proportion of hCD8^+^ T cells having undergone a specified number of cellular divisions by CFSE staining, (**H**) Ki-67 expression, (**I**) TNFα expression, and (**J**) IFN-γ expression on hCD3^+^CD8^+^ T cells. Concatenated flow plots indicate the sum proportion of marker positive cells for all donors in each group. The colored symbol legend is applicable to all comparisons (*n* = 3 to 4 blood donors per group). All plotted data are shown as means with SEM and were analyzed by Brown-Forsythe and Welch ANOVA with Dunnett’s T3 multiple comparisons test or by Kruskal-Wallis with Dunn’s multiple comparisons test.

On the basis of the findings above, PBMCs from EBV^−^ HD were suspected to be less reactive to antigen challenge in the HuPBMC EAE model than EBV^+^ HD and RRMS PBMCs. The observation that FOXP3 positivity among the circulating hCD4^+^ T cell population is reduced even before EAE induction suggests that engrafted xenogeneic T cells may become more inflammatory during reconstitution due to a lack of T_reg_ control, which then transfers to the CNS after immunization. Within the donor PBMCs (before engraftment into NSG mice), hCD4^+^ and CD8^+^ T cells did not differ in their group baseline expression of the activation markers HLA-DR, CD38, CD137, or CD154 (fig. S10) or of subset-specific transcription factors (fig. S11). Likewise, baseline cytokine expression did not follow any EBV- or RRMS-associated trends that would explain the subsequent observations in HuPBMC EAE mice (fig. S11). To evaluate the possibility that T cells from an EBV-exposed host are more intensely activated following T cell receptor (TCR) engagement generally, we stimulated donor PBMCs via hCD3/CD28 engagement in vitro (Fig. 7). Direct stimulation of the TCR without a specific antigen resulted in a trend of enhanced hCD4^+^ T cell proliferation with donor EBV and RRMS status (Fig. 7, A to C, and fig. S12). Although inter-donor variability was high within the baseline measurements, especially in the broader EBV^+^ HD population given the small number of three to four donors per group available for this initial analysis, a trend of increased T cell proliferation with EBV and RRMS status consistently emerged. hCD4^+^ T cells from EBV^+^ HD PBMCs and even more so EBV^+^ RRMS PBMCs were able to attain a higher average number of cellular divisions during the incubation period compared to those from EBV^−^ donor PBMCs (Fig. 7B). Consistent with the HuPBMC EAE model data, these T cells were not differentially polarized after stimulation based on inflammatory cytokine expression (Fig. 7, D and E). Moreover, the same effects were observed with hCD8^+^ T cells, as measured by an incremental increase in proliferation with donor EBV seropositivity and RRMS diagnosis following anti-CD3/CD28 treatment (Fig. 7, F to H, and fig. S12). Stimulated hCD8^+^ T cell cytokine expression also did not follow any group-specific trends (Fig. 7, I and J). Therefore, in addition to an EBV- and RRMS-associated deficiency in the expansion of T_regs_ in the HuPBMC EAE model, both hCD4^+^ and hCD8^+^ T cells from EBV-experienced hosts were more proliferative following ex vivo TCR stimulation, without alteration to the polarization profiles of these cells.

## DISCUSSION

The findings of this study highlight the utility of PBMC-humanized EAE mice in modeling clinical and immunopathological aspects of MS and its association with a history of EBV infection. Immunocompromised NSG mice engrafted with human donor PBMCs and immunized with myelin antigen were susceptible to the development of EAE symptoms and demyelination alike classical murine EAE models. As confirmed by histological analysis, engrafted human CD8^+^ T cells were able to migrate to and infiltrate the mouse CNS and localize to areas of microgliosis and myelin damage. The CNS of symptomatic mice was enriched for hCD8^+^ relative to hCD4^+^ T cells, which is commonly observed in postmortem analysis of MS brain lesions (*57*, *68*) but is not usually observed in classical murine EAE models (*37*). We also observed equivalent numbers of infiltrating human T cells in the brain and the spinal cord, as opposed to the spinal cord dominant pathology of most murine EAE models (*37*). Within the brain, hCD8^+^ T cell infiltration was concentrated in the cerebellum, consistent with murine EAE models where mCD4^+^ T cells are known to infiltrate the vascularized cerebellar tissue (*69*, *70*). Infiltrating T cells produced predominantly T_H_1 and cytotoxic molecules that functioned alongside phagocytic murine myeloid cells to demyelinate the CNS. Notably, demyelination of the mouse CNS occurred without substantial involvement of the few engrafted human macrophages.

Interspecies TCR:major histocompatibility complex (MHC) interactions are potentially less efficient than autologous antigen presentation (*71*), as evidenced by a relatively reduced rate of EAE symptom incidence compared to classical murine EAE models (*72*). These interactions are, however, sufficient to produce demyelinating disease, resulting in clinically measurable motor deficits when HuPBMC mice are immunized with MOG. A previous study incorporated myelin antigen-pulsed autologous dendritic cells (DCs) to provide human MHC for presentation in PBMC humanized mice (*73*), although our data suggest that priming by human DCs was not required to produce clinical symptoms in EAE-induced HuPBMC mice. While we did not observe any differences in xGvHD-specific clinical symptoms between our recipient groups, human T cell responses to the CNS in HuPBMC EAE mice are necessarily xenoreactive because they involve recognition of murine MHC complexes. As autoimmune disease and graft-versus-host disease (GvHD) are both forms of immune-mediated tissue rejection with overlapping characteristics (*74*, *75*), more concrete conclusions about the relative role of myelin-specific T cell reactivity versus nonspecific xGvHD in the HuPBMC EAE model should be assessed in the future by determining the epitopes of MOG protein presented to xenogeneic T cells by NSG-encoded MHC complexes and using more sensitive measures to distinguish graft and myelin-specific human T cell responses. Generally, immune system reconstitution of NSG mice with human PBMCs does not precisely replicate the composition of the donor PBMCs or all the nuanced contextual and site-specific complexities of the mammalian immune system (*62*). Nevertheless, the current data collectively demonstrate that the HuPBMC EAE model is a personalizable, interspecies system that exemplifies key elements of MS and, as such, is a viable mouse model capable of providing clues for causation, biomarkers, and treatment of disease.

PBMC donor history of EBV infection and a diagnosis of RRMS both exacerbated clinical disease susceptibility and severity in HuPBMC EAE mice. Consistent with the findings of Bjornevik *et al.* (*10*), clinical EAE differences could not be attributed to other factors such as CMV infection status or reduced serum vitamin D levels. Clinical differences associated with donor EBV seropositivity in the HuPBMC EAE model are consistent with findings that elevated humoral responses to EBV at disease onset are associated with long-term greater disease severity and higher relapse rate in individuals with MS (*76*). The association between EBV and relapse risk has been suggested to be because of increased EBV-specific CD8^+^ T cell activity and exhaustion during relapse following ineffective immune control of infection during the remission phase (*77*, *78*). Although EBV infection alone was not necessary to enable EAE development in HuPBMC mice, the EBV^+^ HD recipient group presented with an intermediate phenotype that resembled RRMS recipient mice in some ways more than the EBV^−^ HD group. Active infection by EBV was not required to exacerbate disease severity, as demonstrated by undetectable viral replication in the PBMC samples from donors with existing latent EBV infection, as well as a lack of reactivation and memory B cell responses to EBV in mice derived from these donors.

Our group has previously demonstrated that viral reactivation does not occur following EAE induction in C57Bl/6 mice latently infected with the murine homolog of EBV, γHV68, and that γHV68 does not actively infect the CNS during EAE (*35*). B cell accumulation in the CNS of HuPBMC EAE mice was also not significantly different among mice derived from donors with varying EBV and RRMS status. Although relatively few human B cells engrafted and infiltrated the CNS compared to T cells, the possibility remains that engrafted B cells of varying specificities and functionalities, which can act both protectively and pathogenically in classical EAE models (*79*, *80*), may have contributed to the enhancement of disease and the increased CNS inflammation observed in EBV^+^ and RRMS recipient mice. Clinical postmortem evidence has suggested that EBV could contribute to the development and/or progression of MS through infection of CNS-infiltrating B cells and tertiary lymphoid structures, leading to local inflammation (*81*–*83*). With minimal B cell accumulation in the CNS by endpoint, the HuPBMC EAE model likely reflects earlier stages of MS, where B cell–containing lymphoid structures have not yet formed. An important limitation of PBMC humanized mice is the development of clinically overt xGvHD, which limited the duration of experiments to ~25 days postimmunization in this study. Further investigation will be required to assess the presence and influence of EBV-infected and EBV-specific B cells in HuPBMC EAE mice. Although there are multiple supported mechanisms by which EBV could promote an autoimmune environment through B cell–specific processes, our data suggest a role for T cell immunomodulation following latent infection.

The CNS tissues of HuPBMC EAE mice derived from EBV^+^ HD and RRMS recipient groups contained an increased number of T_H_1 and cytotoxic T cells compared to EBV^−^ HD–derived samples. Additionally, EBV^+^ mice also showed substantially reduced hCD4^+^FOXP3^+^ T_reg_ expansion, suggestive of an impairment in immune regulation due to prior infection. The increased abundance of effector T cells relative to T_regs_ consequently skewed to promote a more inflammatory environment in mice derived from HDs with a history of EBV infection, which further compounded when donors were also diagnosed with RRMS. Unlike in classical EAE models, where inbred mice are effectively genetically identical and highly susceptible to EAE immunization (*84*, *85*), the genetic variability of engrafted human immune cells from unrelated donors highlights the consistency and magnitude of the effect of EBV seropositivity on disease outcomes in this model. Given that both clinical and immunological differences were observed between the HD groups that differed only by EBV serostatus, these effects were not entirely due to existing autoimmunity among PBMC donors. The increase in CNS-infiltrating T effector cells appeared to be because of an enhanced proliferative capacity among T cells derived from EBV^+^ donors following TCR engagement, although analysis of greater numbers of PBMC donors than were available herein would be required to establish a more generalizable conclusion. Using a lysophosphatidylcholine model of demyelination in NSG mice, El Behi *et al.* (*86*) noted that engraftment of preexisting lesions with PBMCs obtained from individuals diagnosed with MS impaired remyelination compared to HD PBMC engraftment, consistent with our findings that lymphocytes from patients with MS have “a different intrinsic capacity to respond to stimulation.” Further analysis will be required to determine whether engrafted human T cells that proliferate in the periphery express more CNS homing molecules and chemokine receptors when derived from EBV^+^ donors, or, if upon infiltration of the CNS, EBV-experienced T cells proliferate locally to a greater extent than those that are EBV naïve. T_regs_ control effector T cell proliferation and motility in the CNS following EAE induction (*87*), and, thus, assessment of the functional capacity of the engrafted cytotoxic T cell and T_reg_ populations would also provide insight into mechanisms by which EBV suppresses regulation of xenogeneic effector cells following antigenic challenge.

As T cells are required for constant immune control of EBV-infected B cells (*88*), latent infection could promote lasting change in predisposition to activation that may alter the balance of effector and regulatory responses toward a more “MS-like” state. In the γHV68-EAE model, we also observe greater CD8^+^ T cell infiltration of the CNS and, particularly, the brain of latently infected mice, in addition to T_H_1 skewing, macrophage/microglia and DC activation, and reduced T_reg_ proportions in the CNS and periphery (*35*, *36*). Similar T_H_1 skewing and CD8^+^ T cell involvement was observed in latent γHV68–infected mice with collagen-induced arthritis (*89*). Our findings collectively suggest a common role for gammaherpesviruses in the exacerbation of and predisposition for autoimmune disease, independent of the inciting tissue-specific antigen that initially prompts autoreactivity, through global, systemic skewing of the T cell compartment toward a more proinflammatory T_H_1/cytotoxic and less regulated response upon activation with an autoantigen in a susceptible individual.

Other susceptibility factors for MS, including a history of obesity, smoking, and low vitamin D intake, have all been suggested to act, in part, by promoting T cell inflammation both directly and by impeding regulatory processes (*90*–*93*). Ingelfinger *et al.* (*94*) recently identified increased CD25 expression on transitional helper T cells as a defining phenotype of MS-derived PBMCs in a monozygotic twin study, which the authors posit could be preferentially responsive to an environmental immune challenge such as EBV infection. These findings collectively suggest that the mechanisms for EBV-mediated risk restricted to specific antigens, such as molecular mimicry, are not the only means by which EBV infection could predispose an individual to development of clinically apparent MS. A more generalized pathogenic effect on T cells of varying specificities would also explain the robust link between EBV and other, antigenically distinct autoimmune diseases. Zdimerova *et al.* (*95*) also demonstrated a synergistic interaction between EBV infection and the MS risk allele HLA-DRB1*15:01 in stem cell–humanized mice, leading to increased T cell reactivity to myelin antigens. Although two of the four RRMS donors who provided samples to this study expressed the HLA-DRB1*15:01 risk allele, the absence of the variant among HDs in this small initial donor pool did not allow for an analysis of the downstream effects on disease outcomes. A focus on the interplay of genetics and environmental exposures will enable more comprehensive investigation of MS risk factors and targeted therapies in the HuPBMC EAE model moving forward.

HuPBMC mice offer the possibility of prescreening and selecting PBMC donors based on chosen genetic variants, environmental exposures, immunological conditions (*96*, *97*), and, in the case of the HuPBMC EAE model, MS disease characteristics of interest to create a personalized immune system model. While EBV-induced immunomodulation of T cell responses is one possible mechanism by which infection could incite or promote MS, it is also possible that immunomodulation occurs alongside additional virus-specific mechanisms, such as infection and immortalization of autoreactive B cells, ineffective or aberrant immune control of EBV reactivation, and/or structural mimicry of CNS antigens by viral proteins (*11*, *98*–*101*). An important next step will be to assess clinical outcomes and T cell inflammation in HuPBMC mice engrafted with PBMCs derived from donors with other autoimmune disorders, especially those also linked to EBV infection, such as systemic lupus erythematosus and rheumatoid arthritis (*28*, *29*), to determine the generalizability of T cell immunomodulation by EBV in autoimmune susceptibility. HuPBMC EAE mice could also be used to directly assess potential disease-modifying therapies specific to human immune targets, as well as indirectly by engrafting PBMCs derived from patients treated with such therapies like B cell–depleting monoclonal antibodies or EBV-specific T cells (*102*). Instances of promising results from biologic testing in preclinical EAE models that translate to worsened disease or serious side effects in humans could ideally be minimized by using preclinical models with human immune systems before advancement to clinical trials (*103*–*106*). HuPBMC mice could also be used to circumvent the generation of costly transgenic mouse lines for immunogenetic studies. Given the ever-increasing availability of new immunocompromised mouse strains and the many reports of humanization protocols and analytical methods being generated and improved upon (*107*–*110*), specific aspects of disease pathogenesis could be readily assessed using specially adapted humanized EAE model variants. The HuPBMC EAE model mechanistically reflects the epidemiological and clinical data associating EBV infection with increased risk of autoimmune disease.

In summary, we have shown that T cells derived from EBV-infected people, as well as those with a diagnosis of RRMS, are capable of mediating enhanced clinical symptoms and xenogeneic CNS immunopathology in a new human T cell transfer model of EAE compared to T cells from uninfected people. This effect was observed in the absence of an active EBV infection, indicating that lytic reactivation is not the only means by which EBV could pathogenically modulate an autoimmune response in the CNS. We also demonstrated an enhanced proliferative capacity of both hCD4^+^ and hCD8^+^ T cells derived from EBV-exposed donors following direct TCR stimulation without a specific antigen, which links epidemiological and clinical data demonstrating an EBV-related risk for multiple autoimmune diseases with distinct target tissues. Our findings highlight that EBV-mediated risk for disease in a humanized mouse model of MS occurs before antigenic challenge and that either the establishment or maintenance of latent infection modulates subsequent T cell reactivity years later. Because EBV is a potentially preventable infection, these findings have implications for reducing MS incidence by targeting EBV and its downstream effects on host immune responses. Therapeutic interventions aimed at reducing the proinflammatory T cell response prompted by EBV could effectively diminish disease activity in MS and other antigenically distinct EBV-associated autoimmune disorders.

## MATERIALS AND METHODS

### Human participants

Blood donation by human participants was approved by the University of British Columbia’s Clinical Research Ethics Board and by the Fraser Health Authority, under protocol H16-02338. Individuals with RRMS were recruited at the Fraser Health MS Clinic (Burnaby, BC) under the supervision of G. Vorobeychik. Unaffected, otherwise HDs were recruited at the Life Sciences Center (University of British Columbia). All donors were female, were 19 to 39 years of age (mean age of 31.5 ± 6.1 years for RRMS donors and 26.4 ± 6.2 years for HDs), and provided written informed consent before enrolment in the study from November 2018 to August 2021. Donors with a definite RRMS diagnosis, according to Poser or 2010 McDonald criteria, and disease duration of less than 10 years were confirmed as treatment naïve before donation (no previous use of any disease-modifying therapies during lifetime). RRMS donors underwent a neurological exam the day of blood donation to assess expanded disability status scale (EDSS) score. Individuals with a progressive MS diagnosis or EDSS of >4 who were male, pregnant, outside of the designated age range, or undergoing treatment were excluded from the present study.

### Donor sample processing

Blood samples were obtained by venipuncture and assigned an alphanumeric code to protect donor identity. Serum was obtained by centrifugation of blood (15 min at 1300*g*) collected in uncoated vacutainers (BD, no. 367820) and frozen at −80°C before analysis. Whole blood (80 ml) was processed for PBMC isolation by Lymphoprep (STEMCELL Technologies, no. 07801) gradient separation, according to the manufacturer’s instructions, within an hour of collection in K_2_-EDTA–coated vacutainer tubes (BD, no. 366643). Donor PBMCs were immediately injected into recipient mice (not frozen prior). A subsample of freshly isolated PBMCs was retained for DNA isolation and flow cytometric analysis. A second subsample of PBMCs was stored in autologous plasma with 10% dimethyl sufoxide and placed in a Mr. Frosty Freezing Container (Thermo Fisher Scientific, no. 5100-0001) for controlled cooling to −80°C, followed by transfer to liquid nitrogen for long-term storage. DNA samples extracted from donor PBMCs were HLA genotyped by The Sequencing Center (Fort Collins, Colorado, USA) using GenDx NGSengine software for HLA typing (v.2.27.1).

### Animals

Animal work was approved by the Animal Care Committee of the University of British Columbia, under regulation of the Canadian Council of Animal Care, under protocols A17-0266 and A17-0184. Adult male NSG (NOD.Cg-*Prkdc^scid^ Il2rg^tm1Wjl^*/SzJ, JAX no. 005557), NSG-SGM3 [NOD.Cg-*Prkdc^scid^Il2rg^tm1Wjl^*Tg (CMV-IL3,CSF2,KITLG)1Eav/MloySzJ, JAX no. 013062], and NOD (JAX no. 001976) mice, originally sourced from the Jackson Laboratory, began experiments at 6 to 14 weeks old. In preliminary experiments, we observed a greater incidence of xGvHD and reduced EAE symptom incidence in female NSG mice compared to that in male NSG mice engrafted with the same donor PBMCs (fig. S13), and, therefore, male NSG mice were used as recipients for this study. Moreover, NSG-SGM3 mice, which differ from the NSG by transgenic expression of human hematopoietic cytokines (*67*, *111*), exhibited similar incidence of EAE and xGvHD symptoms as NSG mice engrafted with the same donor PBMCs (fig. S14) and were thus used interchangeably when randomized to recipient groups. Mice were bred in three facilities in Vancouver, British Columbia (BC Cancer Animal Resource Centre, Centre for Disease Modelling, and Modified Barrier Facility), to minimize any potential facility-specific outcomes [as has been previously reported to be a factor in EAE studies (*112*, *113*)] and housed in the same specific pathogen–free facility at the University of British Columbia for the duration of experiments (Modified Barrier Facility). Mice were housed in groups up to five animals per cage on corn cob bedding (Bed-o’Cobs, The Andersons) and fed ad libitum with PicoLab Rodent Diet 20 (no. 5053) standard irradiated chow with free access to autoclaved acidified water (pH 3 to 4). Housing rooms were set at 22° to 25°C with a humidity range of 50 to 70%. All NSG cages were kept on the same designated immunocompromised rack on a 14.5-hour light–9.5-hour dark cycle with sunrise at 5:30 a.m. and sunset at 8:00 p.m.

### Humanization and EAE induction

NSG mice were engrafted with 5 × 10^6^ donor PBMCs each [or blank phosphate-buffered saline (PBS) for un-engrafted controls] by intravenous tail vein injection. Twelve to 20 mice per donor were randomly assigned to recipient groups on the basis of PBMC yield and availability on the day of blood donation. Following a 3-week reconstitution period, circulating human CD45^+^ cell repopulation was confirmed by saphenous vein blood sampling and flow cytometric analysis. HuPBMC mice, as well as un-engrafted NSG and NOD control mice for some experiments, were actively induced with EAE on day 0 by subcutaneous injection of a 100-μl total volume containing 200 μg of MOG_35-55_ peptide (GenScript), 100 μg of recombinant human MOG (rhMOG_1-120_ prepared in-house), and 400 μg of desiccated *Mycobacterium Tuberculosis H37Ra* (DIFCO, no. DF3114-338) emulsified in incomplete Freund’s adjuvant (DIFCO, no. DF0639-60-6). On days 0 and 2 postimmunization, mice also received an intraperitoneal injection of 200 ng of pertussis toxin (List Biological Laboratories, no. 179A). The mice were monitored and scored daily for EAE symptoms. Clinical EAE scoring was based on the following 5-point scale: 0, no overt signs of disease; 0.5, partially limp tail; 1, limp tail; 1.5 limp tail and hind limb weakness; 2, loss of coordinated movements; 2.5, one hind limb paralyzed; 3, both hind limbs paralyzed; 3.5, hind limbs paralyzed and weakness in forelimbs; 4, forelimbs paralyzed; and 5, moribund state or death by EAE. Clinical outcome assessors of HuPBMC EAE mice were blinded to the viral serostatus of the blood donors. Onset was defined by the occurrence of tail paralysis (score 0.5 or higher) for two consecutive days. Cumulative EAE scores were calculated by summing daily clinical scores from the day of onset until endpoint. Mice with motor impairments, weight loss, and signs of illness were provided supportive care, including administration of subcutaneous fluids and cage heating, and provisions of wet food and nutritional gel. Humane endpoint was defined by an EAE score of 4 or greater or a clinical health score of 4 or greater, as defined by the Animal Care Committee based on body condition, weight loss, and signs of pain. Symptomatic onset of xGvHD was defined by the appearance and persistence or worsening of skin dryness, redness, discoloration (jaundice), and/or hair loss. Because of intragroup variability in EAE symptom onset and ongoing xGvHD and wasting following the resolution of EAE symptoms, HuPBMC EAE mice could not be maintained longer than ~10 days post–symptom onset. Recipient cohorts were treated with the same experimental interventions for humanization and EAE induction and assigned at random to different endpoint analyses.

### Recombinant human MOG protein

The extracellular domain of recombinant human myelin oligodendrocyte protein (rhMOG_1-120_) was expressed from an *Escherichia coli* vector (pQE-30) obtained from C. Linington and N. Ruddle (*114*, *115*), and the protocol was modified from that of J. Gommerman. The vector expresses a 15.8-kDa His-tagged rhMOG protein under the control of a lac operon. Following isopropyl ß-d-thiogalactopyranoside induction, the cells were lysed to release the protein product from inclusion bodies. The protein was subsequently purified using a Ni^2+^-His-bind resin column (5-ml HisTrap FF; Cytiva, no. 17525501), and the protein fractions were analyzed by SDS–polyacrylamide gel electrophoresis gel electrophoresis and stained with Coomassie blue. The fractions containing sufficiently pure protein of the correct molecular weight were pooled and diluted to a protein concentration of <0.5 mg/ml in preparation for partial refolding in cellulose dialysis tubing [6- to 8-kDa molecular weight cutoff (MWCO)]. The refolded protein product was concentrated by centrifugation in 3-kDa MWCO Amicon tubes (EMD Millipore, no. UFC900324) until a final concentration of at least 4 mg/ml was reached. The final concentration was determined using a Nanodrop Lite Spectrophotometer (Thermo Fisher Scientific, firmware version 1.02) (*M*_w_ = 15.5 kDa, *e*/1000 = 12.09). Protein samples were aliquoted and flash frozen in liquid nitrogen before storage at −80°C. The purified and concentrated rhMOG protein was confirmed to elicit an encephalitogenic response by induction of EAE symptoms in C57Bl/6 mice.

### Tissue collection

Mice were euthanized at endpoint by isoflurane overdose. Blood was collected by cardiac puncture, followed by perfusion with 20 ml of sterile PBS for collection of CNS tissues. The brains, spinal cords, and spleens were dissected whole and kept in cold sterile PBS or in 10% neutral-buffered formalin (NBF) for further processing. Spinal cords were straightened, placed onto filter paper, and submerged in NBF for histological analyses. The presence of intact emulsion was confirmed by examining the skin at the injection site. Serum was extracted from untreated blood samples by centrifugation (13 min at 8000 rpm) and stored at −80°C before analysis. To isolate peripheral blood cells, collected blood samples were treated with K_2_-EDTA for further processing. Tissues from animals in each recipient cohort were included for analysis regardless of the occurrence of clinically visible EAE symptoms to account for differences in incidence and severity among donor groups.

### Histological analysis

#### Immunohistochemistry

Perfused, intact organs collected for analysis of cellular infiltration by immunohistochemistry were stored in 10% NBF for 2 hours at room temperature (RT). At 2 hours, brains were halved by sagittal division and incubated for another 2 hours along with the spinal cords (4 hours total in NBF at RT). CNS organs were washed in cold PBS for 1 hour at 4°C to remove remaining NBF and then placed in 15% sucrose solution overnight at 4°C for cryoprotection. Organs were then placed in a 30% sucrose solution overnight at 4°C and then placed overnight at 4°C in a 25% sucrose solution containing 50% optimal cutting temperature (OCT) compound (VWR Clear Frozen Section Compound, no. CA95057-838). Cryoprotected organs were segmented in the case of spinal cords and oriented in cryomolds containing OCT medium and frozen on a partially submerged aluminum block equilibrated in liquid nitrogen and then stored at −80°C for sectioning. Brain and spinal cord tissues were cut at −19°C into 12-μm sagittal and coronal sections, respectively, using a Shandon Cryotome FSE (Thermo Fisher Scientific), and placed onto Superfrost Plus microscope slides (Thermo Fisher Scientific, no. 12-550-15). Sections were taken at least 30-μm apart and obtained from one of either brain hemispheres or from four equally distant points along the length of the spinal cord. Sections were rehydrated in PBS for 10 min at RT, followed by blocking in 3% mouse serum in PBS overnight at 4°C. Sections were washed thrice in PBS (5 min per wash) and incubated with rabbit antihuman CD8 primary antibody (stock diluted 1:200; table S1) and/or goat anti-mouse/human Iba-1 primary antibody (0.5 mg/ml, stock diluted 1:300) in 3% mouse serum in PBS for 2 hours at 4°C. A rat IgG isotype primary (Abcam, no. ab27478) was used to confirm the specificity of the secondary antibody binding to antihuman CD8. Sections were then washed thrice and incubated with Alexa Fluor 680–conjugated donkey anti-rabbit IgG secondary antibody (2 mg/ml, stock diluted 1:600) or DyLight594-conjugated mouse anti-goat IgG secondary antibody (1 mg/ml, stock diluted 1:300) in 3% mouse serum in PBS for 2 hours at 4°C. Sections were washed thrice with PBS and then incubated for 20 min at RT in PBS containing 0.2% Triton X-100 (PBT solution). CNS sections were incubated 20 min at RT in a PBT master mix containing FluoroMyelin Green, NeuroTrace 530/615, and 4′,6-diamidino-2-phenylindole (Brain-Stain Imaging Kit, Invitrogen, no. B34650). Sections were then washed thrice in PBT (15 min per wash) to remove excess stain. Slides were mounted with ProLong Diamond Antifade Mountant medium (Invitrogen, no. P36961), sealed, allowed to cure for 24 hours at RT, and then stored at 4°C until imaged.

#### Demyelination

Perfused, intact spinal cords collected for analysis of myelination were stored in 10% NBF overnight at RT and then moved to 70% ethanol and sent to Wax-it Histology Services Inc. (Vancouver, Canada) for paraffin embedding. To quantify myelination throughout the organ, a total of six to nine consecutive 10-μm coronal sections were taken from four to six equally distanced regions along the length of the spinal cord using a Shandon Finesse 325 microtome (Thermo Fisher Scientific). Sections were mounted on Superfrost Plus microscope slides (Thermo Fisher Scientific, no. 12-550-15), dried at 37°C for 1 hour, and then air dried overnight at RT. Sections were washed in three changes of xylene for 10 min each, then treated in three changes of absolute ethanol for 3 min each, and hydrated in 95% ethanol for 5 min. Slides were incubated in eriochrome cyanine R (EC, Sigma-Aldrich, no. 3564-18-9) staining solution (0.22% w/v ferric chloride, 0.5% v/v sulfuric acid, and 0.2% w/v eriochrome cyanine R CI 43820) for 20 min, run under tap water for 30 s, and incubated in differentiating solution (5.6% w/v ferric chloride) for 5 to 10 min until only the white matter retained the stain. Slides were washed under running tap water for 5 min, then dehydrated in 3 changes of 100% ethanol for 30 s each (with agitation), and cleared in three changes of xylene for 30 s each (with agitation). Coverslips were mounted with VectaMount Permanent Mounting Medium (Vector Laboratories, no. H-5000).

#### Imaging and analysis

Histology sections were imaged using a Zeiss Axio Observer 7 epi-fluorescent microscope outfitted with a motorized stage (*x*, *y*, and *z* mobility) and five light-emitting diode (LED) channels. Whole-organ immunofluorescent sections were imaged at 20×/0.65 numerical aperture (NA) air objective using an Axiocam 702 mono complementary metal-oxide semiconductor (CMOS) camera at RT. Isotype staining was used to set LED voltages and exposures across slides per imaging experiment. Immunofluorescent images were stitched, and *Z*-stack (three stacks of 1.2 μm each) fusion was performed using a wavelet transform in Zen2.6 Pro (blue edition, Zeiss). Bright-field images of EC-stained spinal cord sections were acquired at 10×/0.3 NA air objective magnification using an Axiocam 105 color CMOS camera. Stitching and subsequent analysis of images was performed using Zen 2.6 Pro. For EC sections, single RBG images were saved as JPEG files, and quantitative analysis of staining was performed using ImageJ 1.53c (NIH). Default color thresholding method was used to first select the area of stained myelin and then the total area of the spinal cord. Hue, saturation, and brightness thresholds were set on the basis of fully myelinated NSG control sections and applied across all other sections. The area measurement function was used to acquire the area of the selected regions in square micrometers, relative to a 200-μm scale bar. Myelination was expressed as fraction of myelin-stained area of total area of the spinal cord section. Replicate sections (six to nine consecutive sections per region) were averaged to generate four to six regional myelination indices per spinal cord.

### Flow cytometric analysis

#### Sample processing

The brains, spinal cords, and spleens were kept in sterile PBS on ice and then processed into single-cell suspensions by passing the tissue through a 70-μm cell strainer with a syringe insert. Splenocyte suspensions were incubated in red blood cell lysis buffer (150 mM NH_4_Cl, 10 mM KHCO_3_, and 0.1 mM Na_2_-EDTA) for 10 min on ice. Whole EDTA-treated blood samples were incubated in pre-warmed red blood cell lysis buffer for 15 min at RT. Brain and spinal cord samples specifically processed for the detection of intracellular MBP within phagocytic cells were predigested with collagenase/dispase (0.5 mg/ml; Roche, no. 11097113001), deoxyribonuclease I (0.02 mg/ml; Sigma-Aldrich, no. D5025), and 2% fetal bovine serum (FBS) in 10 ml of RPMI 1640 per sample for 45 min of shaking at 180 rpm and 37°C. CNS cells were further isolated by resuspension in 40% isotonic Percoll solution (Cytiva, no. 17089101) and centrifugation (15 min at 1400 rpm) to remove lipid debris. Samples analyzed for cytokine expression were incubated at 37°C (5% CO_2_) for 4 hours at 1 × 10^6^ to 2 × 10^6^ splenocytes or total isolated CNS cells per 200 μl of stimulation medium, containing 10% FBS, phorbol 12-myristate 13-acetate (PMA; 10 ng/ml; Sigma-Aldrich, no. P1585), ionomycin (500 ng/ml; Thermo Fisher Scientific, no. I24222), and GolgiPlug Protein Transport Inhibitor (1 μl/ml; containing Brefeldin A; BD Biosciences, no. 555029). Cell suspensions were washed between each step with sterile PBS containing 2% FBS [fluorescence-activated cell sorting (FACS) buffer].

#### T cell bead stimulation assay

Donor PBMC samples previously stored in autologous plasma with 10% dimethyl sulfoxide were thawed from liquid nitrogen for activation analysis. Total PBMCs (5 × 10^6^ cells/ml) were stained with 2.5 mM carboxyfluorescein succinimidyl ester (CFSE; Sigma-Aldrich, no. 21888-25MG-F) in the dark at 4°C for 8 min before quenching with newborn calf serum. An unstimulated and PMA/ionomycin-stimulated subset of each sample was analyzed for baseline marker expression (PMA stimulation conditions described in the previous section). Duplicate wells of 200,000 CFSE-stained cells per sample were then incubated for 91 hours at 37°C (5% CO_2_) at a 1:2 bead-cell ratio with Human T-Activator CD3/CD28 DynaBeads (Thermo Fisher Scientific, no. 11161D) and recombinant human IL-2 (1 U/ml; BioLegend, no. 589102) in 200 μl of complete culture medium [RPMI 1640 and 10% FBS, 2 mM l-glutamine, penicillin/streptomycin (50 U/ml), 1× MEM with non-essential amino acids (NEAAs), 1 mM sodium pyruvate, 10 mM Hepes, and 50 μM β-mercaptoethanol]. The medium did not contain any factors to specifically influence polarization or activation. Cells were then refreshed with pre-warmed culture medium containing GolgiPlug Protein Transport Inhibitor (1 μl/ml) and incubated for another 5 hours at 37°C (5% CO_2_). Following 96 hours total incubation time, cells were washed with serum-free PBS and stained for flow cytometric analysis as described below.

#### Antibody staining and analysis

Cells were stained with 1× eBioscience Fixable Viability Dye eFluor 506 (Thermo Fisher Scientific, no. 65-0866-14) for 20 min at 4°C in serum-free PBS. Cells were then washed in FACS buffer and preincubated with human and mouse Fc block (table S1) in FACS buffer for 10 min at RT. Cells were washed again and incubated with fluorochrome-labeled antibodies specific to extracellular makers (table S1) in FACS buffer for 30 min at 4°C. Cells were washed and treated with transcription factor fixation and permeabilization reagent (Thermo Fisher Scientific, no. 00-5521-00) for 30 min at 4°C, followed by a wash with permeabilization buffer (Thermo Fisher Scientific, no. 00-8333-56). Cells were incubated with fluorochrome-labeled antibodies specific to intracellular makers (table S2) in permeabilization buffer for 30 min at RT. Cells were washed with permeabilization and FACS buffer and resuspended in FACS buffer with 2 mM EDTA for acquisition. Frozen human PBMC samples were stained as detailed above for use as titration, compensation, and gating controls for all experiments. Stained cell suspensions were acquired on an Attune NxT flow cytometer (Thermo Fisher Scientific) and analyzed using FlowJo v10.8 Software (BD Life Sciences).

### Serology

Endogenous antibodies to EBV, CMV, and rhMOG were detected using indirect enzyme-linked immunosorbent assays (ELISAs). Nunc Maxisorp 96-well microtiter ELISA plates (Thermo Fisher Scientific, no. 439454) were coated overnight at 4°C with 1 μg per well of the peptide or protein of interest (table S3, produced by ScenicBio) in 0.05 M carbonate buffer. For EBV EBNA-1 and CMV, epitope peptides 1 and 2 were mixed before well coating. The following day, the plates were washed thrice with PBS and wash buffer (PBS and 0.05% Tween 20), followed by a 2-hour blocking step at RT using wash buffer and 3% bovine serum albumin. Plates were washed again and then incubated with serum samples serially diluted in blocking buffer to generate duplicate 6-point curves (1:100 to 1:5000 for human donor serum and 1:50 to 1:2500 for mouse serum). After a 2-hour RT incubation with serum dilutions, the plates were washed and then incubated with horseradish peroxidase–labeled antihuman IgG antibody (table S1) diluted 1:3000 in blocking buffer or HRP-labeled antihuman IgM diluted 1:4000 for 1 hour at 37°C. The plates were then washed with PBS before the addition of trimethylboron substrate (100 μl per well; BD Biosciences, no. 555214). Fifteen minutes after the addition of substrate, 100 μl of stop solution (1 M sulfuric acid) was added to each well. The plates were read at 450 nm on a VarioSkan Plate Reader (Thermo Fisher Scientific) within 10 min of adding stop solution. Donor serum at 1:1000 dilution was used to determine seropositivity to EBV and CMV antigens. The lower limit of detection for positive IgG and IgM values was set on the basis of the level of nonspecific background signal in negative control samples (full reaction minus antigen and/or serum). Donor serum vitamin D levels were quantified using a 25-OH Vitamin D ELISA assay test kit, according to the manufacturer’s instructions (Eagle Biosciences, no. VID31-K01).

### EBV viral load

Genomic DNA was isolated from a maximum of 4 × 10^6^ donor PBMCs or mouse splenocytes per sample using the PureLink Genomic DNA Minikit (Invitrogen, no. K1820), according to the manufacturer’s instructions, quantified using a Nanodrop Lite Spectrophotometer (Thermo Fisher Scientific, firmware version 1.02), and stored at −80°C. A *BALF5* viral DNA polymerase gene quantitative polymerase chain reaction (qPCR) protocol adapted from (*50*) was used to measure EBV load in DNA samples. The forward and reverse primer and fluorogenic probe sequences are listed in table S4 (produced by Integrated DNA Technologies), along with the *BALF5* gene fragment sequence used for standard curve generation. qPCR reactions comprised 300 ng of genomic DNA, 0.4 μM each primer, 0.2 μM probe, and 1× QuantiNova Probe PCR master mix (QIAGEN, no. 208256). An EBV^+^ B95-8 cell line extract (100 ng per reaction (American Type Culture Collection, no. CRL-1612) was used as a positive control for EBV detection. Blank water was used in negative control reactions. An 8-point standard curve was generated with 1 to 10^7^ *BALF5* gBlock copies per reaction. Reactions were made up in Mx3000P 96-well plates (Agilent Technologies, no. 401333), and data were acquired using a CFX96 Real-Time System C1000 Thermal Cycler (Bio-Rad) and CFX manager 3.1 software for HEX detection. The cycler parameters were set according to the QIAGEN QuantiNova Probe PCR protocol for 2 min at 95°C to activate DNA polymerase and then 45 repeats of 5 s at 95°C and 5 s at 60°C. Sample copies per reaction were determined by interpolation of the standard curve using triplicate-averaged reaction threshold values (*C*_T_), which were then used to calculate viral load. The lower limit of detection was determined by interpolating the *C*_T_ value for the lowest detectable standard point per plate and was normally ~30 EBV copies/μg of DNA.

### Statistical analyses

Data were collated using Microsoft Excel, then graphed, and statistically analyzed using GraphPad Prism software 9.2.0 (GraphPad Software Inc.). Figure panels were composed in Adobe Illustrator V24.3. Most graphs present group SEM or SD unless otherwise stated in the figure legend. No statistical methods were used to predetermine sample sizes, as donor PBMC yield limited recipient cohort size. No inclusion or exclusion criteria were used for analyses, and groups include all mice from the cohort regardless of the incidence of clinical EAE symptoms, unless otherwise stated. Two normally distributed groups of data were analyzed by two-tailed, unpaired *t* test with Welch’s correction. For three or more groups, normally distributed data were analyzed by ordinary one-way analysis of variance (ANOVA) with Tukey’s multiple comparisons test or Brown-Forsythe and Welch ANOVA with Dunnett’s T3 multiple comparisons test. If group data did not pass the Kolmogorov-Smirnov (KS) normality test (or Shapiro-Wilk test when group *N* is too small to compute KS distance), a nonparametric Mann-Whitney test or Kruskal-Wallis with Dunn’s multiple comparisons test was used. A log-rank (Mantel-Cox) test was used for incidence curve analysis, simple linear regression analysis for cell count correlations, and ordinary two-way ANOVA for group comparisons with two variables (i.e., EAE scores over time and ELISA absorbance readings with serum dilution), where the column factor *P* value is reported. Specific statistical tests used for each assay are noted in the figure legends, along with the number of animals or replicates per group. Significance is indicated by asterisks: *P < 0.05, **P < 0.01, ***P < 0.001, and ****P < 0.0001.

## Supplementary Materials

### This PDF file includes:

Supplementary Text Figs. S1 to S14 Tables S1 to S4 Abbreviations
