# Supplementary material for: Epstein-Barr virus infection promotes T cell dysregulation in a humanized mouse model of multiple sclerosis
Source: Sci Adv. 2025 Mar 5;11(10):eadu5110. doi: 10.1126/sciadv.adu5110 (PMC11881922; doi:10.1126/sciadv.adu5110)
Supplement: Supplementary file 1 — Supplementary Text Figs. S1 to S14 Tables S1 to S4 Abbreviations [file sciadv.adu5110_sm.pdf]

Science Advances
AAAS

Supplementary Materials for

Epstein-Barr virus infection promotes T cell dysregulation in a humanized mouse model of multiple sclerosis

Jessica R. Allanach *et al.*

Corresponding author: Marc S. Horwitz, mhorwitz@mail.ubc.ca

Sci. Adv. **11**, eadu5110 (2025)
DOI: 10.1126/sciadv.adu5110

This PDF file includes:

Supplementary Text
Figs. S1 to S14
Tables S1 to S4
Abbreviations

SUPPLEMENTARY TEXT

The supplemental material included with this manuscript contains additional data cited in the text relating to the main figures, pertaining to blood donor serology and immunophenotyping, HuPBMC EAE model characterization, auxiliary recipient group comparisons, and donor T cell activation, polarization, and proliferation. Additional information regarding reagents and abbreviations mentioned in the main text is also included.

SUPPLEMENTARY FIGURES

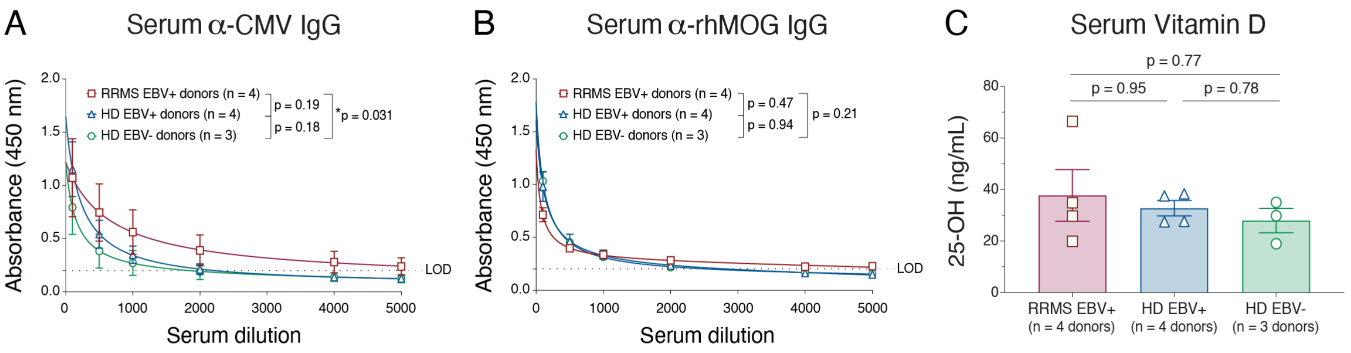

D

Donor HLA Genotyping

Donor ID	HLA-I			HLA-II							
	HLA-A	HLA-B	HLA-C	DRB1	DRB3	DRB4	DRB5	DQB1	DQA1	DPB1	DPA1
MS-01	02:01:01:01<br>11:01:01:01	41:01:01:01<br>50:01:01:01	06:02:01:02<br>17:01:01:05	03:01:01:01<br>03:01:01:01	02:02:01:01<br>03:01:01:01	--<br>--	--<br>--	02:01:01:01<br>02:01:01:01	05:01:01:03<br>05:01:01:03	14:01:01:01<br>104:01:01:01	01:03:01:02<br>02:01:01:01
MS-02	02:01:01:01<br>02:01:01:01	08:01:01:02<br>08:01:01:02	07:02:01:01<br>07:02:01:01	03:01:01:01<br>03:01:01:01	--<br>--	--<br>--	--<br>--	02:01:01:01<br>02:01:01:01	05:01:01:03<br>05:01:01:03	04:01:01:01<br>04:01:01:01	01:03:01:02<br>01:03:01:04
MS-03	02:01:01:01<br>68:02:01:01	07:02:01:01<br>14:02:01:01	05:01:01:02<br>08:02:01:01	13:03:01:01<br>15:01:01:01	01:01:02:01<br>--	--<br>--	01:01:01:01<br>--	03:01:01:03<br>06:02:01:01	01:02:01:01<br>05:05:01:13	02:01:02:01<br>04:01:01:01	01:03:01:01<br>01:03:01:02
MS-04	02:01:01:18<br>26:01:01:01	07:02:01:01<br>35:03:01:01	04:01:01:01<br>07:02:01:03	11:15:01:01<br>15:01:17	--<br>--	--<br>--	--<br>--	03:01:01:03<br>06:02:01:01	01:02:01:01<br>05:05:01:01	02:01:02:01<br>02:01:02:10	01:03:01:01<br>01:03:01:04
HD-01	02:01:01:01<br>11:01:01:01	08:01:01:01<br>44:02:01:01	05:01:01:02<br>07:01:01:01	03:01:01:01<br>13:01:01:01	01:01:02:01<br>02:02:01:02	--<br>--	--<br>--	02:01:01:01<br>06:03:01:01	01:03:01:02<br>05:01:01:02	01:01:01:01<br>19:01:01:01	02:01:02:02<br>02:07:01:01
HD-02	01:01:01:01<br>01:01:01:01	08:01:01:01<br>15:17:01:01	07:01:01:01<br>07:01:02:01	01:01:01:01<br>13:03:01:01	--<br>--	--<br>--	--<br>--	03:01:01:03<br>05:01:01:03	01:01:01:01<br>05:05:01:13	03:01:01:01<br>04:02:01:02	01:03:01:03<br>01:03:01:05
HD-03	01:01:01:01<br>26:01:01:01	08:01:01:01<br>44:03:01:01	07:01:01:01<br>16:01:01:01	03:01:01:01<br>07:01:01:01	--<br>--	01:01:01:01<br>--	--<br>--	02:01:01:01<br>02:02:01:01	02:01:01:01<br>05:01:01:02	04:01:01:01<br>04:01:01:01	01:03:01:02<br>01:03:01:02
HD-04	01:01:01:01<br>32:01:01:01	08:01:01:01<br>40:02:01:01	02:02:02:01<br>07:01:01:01	03:01:01:01<br>14:01:01	--<br>--	--<br>--	--<br>--	02:01:01:01<br>05:03:01:01	01:04:01:01<br>05:01:01:02	03:01:01:01<br>04:01:01:01	01:03:01:01<br>01:03:01:03
HD-05	24:02:01:01<br>24:02:01:01	48:03:01:01<br>54:01:01:01	01:02:01:01<br>08:01:01:01	09:01:02:01<br>11:01:01:01	02:02:01:03<br>--	01:03:02<br>--	--<br>--	03:01:01:03<br>03:03:02:02	03:02:01:01<br>05:05:01:01	05:01:01:01<br>05:01:01:01	01:03:01:01<br>02:02:02:01
HD-06	01:01:01:01<br>02:01:01:01	08:01:01:01<br>40:01:02:01	03:04:01:01<br>07:01:01:01	07:01:01:01<br>08:03:02:01	--<br>--	01:03:01:01<br>--	--<br>--	02:02:01:01<br>03:01:01:17	02:01:01:01<br>06:01:01:03	04:01:01:01<br>06:01:01:01	01:03:01:02<br>01:03:01:03
HD-07	02:01:01:01<br>31:01:02:01	27:05:02:01<br>44:02:01:01	02:02:02:01<br>05:01:01:02	12:01:01:01<br>13:02:01:02	02:02:01:06<br>03:01:01:01	--<br>--	--<br>--	03:01:01:05<br>06:04:01:01	01:02:01:04<br>05:05:01:03	02:01:02:01<br>03:01:01:01	01:03:01:01<br>01:03:01:03

Each cell contains alleles 1 and 2, if applicable

Figure S1.

Donor serology and HLA allele sequencing for confounding MS risk factors. Figure shows donor serum IgG specific to (A) cytomegalovirus (CMV) antigens and (B) the inducing antigen, recombinant human myelin oligodendrocyte glycoprotein ($rhMOG_{1-120}$). In A and B, group data are shown as mean with SEM (n = 3 – 4

donors/group) and were curve fit with a one-site total binding equation. Statistical differences in titre curves were assessed by ordinary two-way ANOVA. The lower limit of detection (LOD) is represented by a dashed line. (C) Donor serum 25-OH vitamin D levels are shown as mean with SEM (n = 3 – 4 donors/group) and were analyzed by Brown-Forsythe and Welch ANOVA with Dunnett's T3 multiple comparisons test. (D) Individual human leukocyte antigen (HLA) genotypes showing alleles sequenced at three HLA-I and eight HLA-II encoding loci for all PBMC donors.

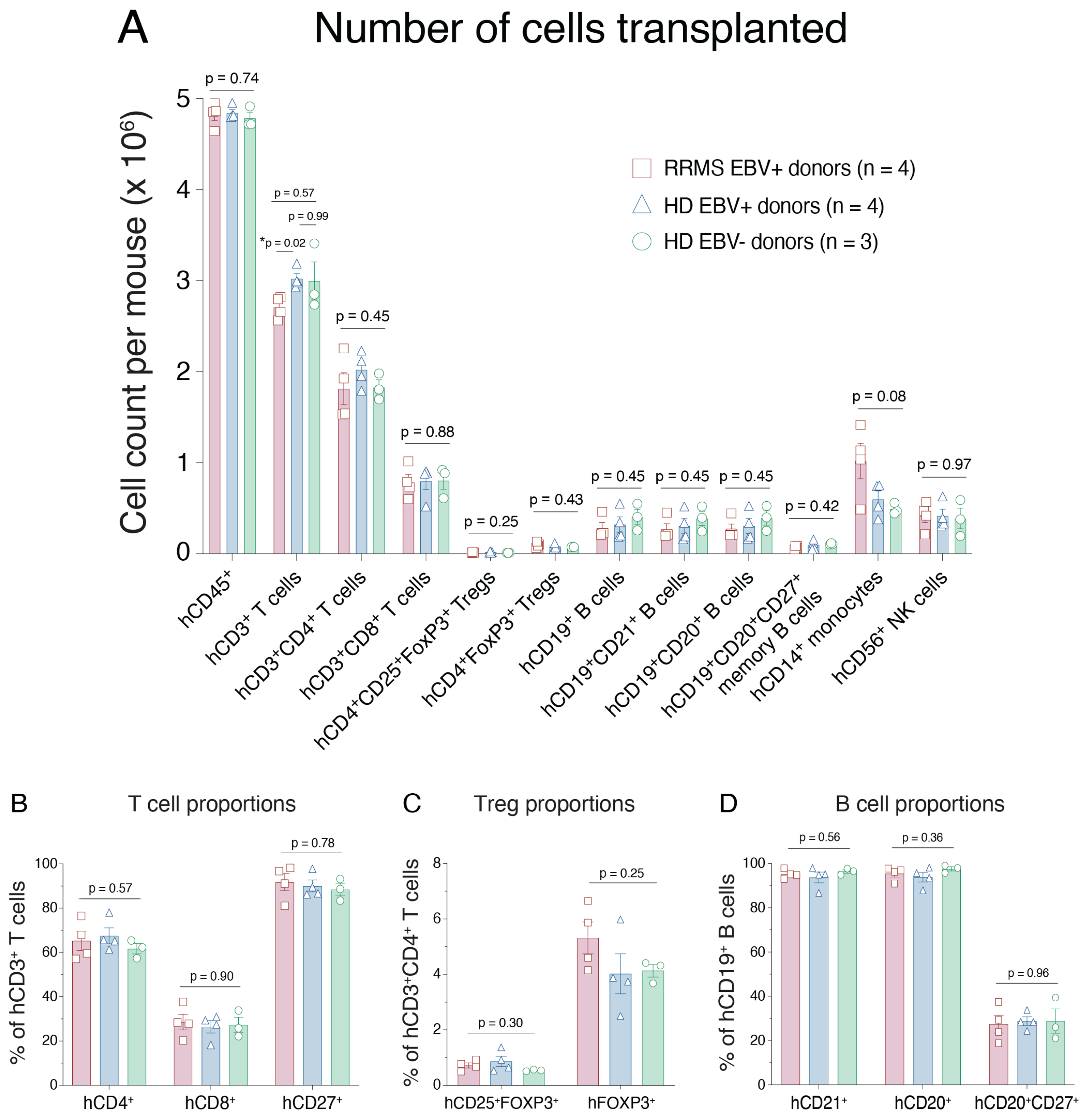

Figure S2.

Phenotypic composition of donor PBMCs injected into recipient NSG mice. Figure shows (A) the number of cells from each major human immune subset transplanted per NSG mouse and the relative proportions of (B) T cell, (C) hCD4$^+$ regulatory T cell (Treg), and (D) B cell subsets among those transplanted cells. In A – D, data are shown as mean with SEM (n = 3 – 4 donors/group) and were analyzed by Brown-Forsythe and Welch ANOVA with Dunnett's T3 multiple comparisons test or Kruskal-Wallis with Dunn's multiple comparisons test.

Nonsignificant p values represent the overall test result for the three-group comparison, wherein each individual group comparison was also determined to be nonsignificant. The symbol legend in A is applicable to B – D.

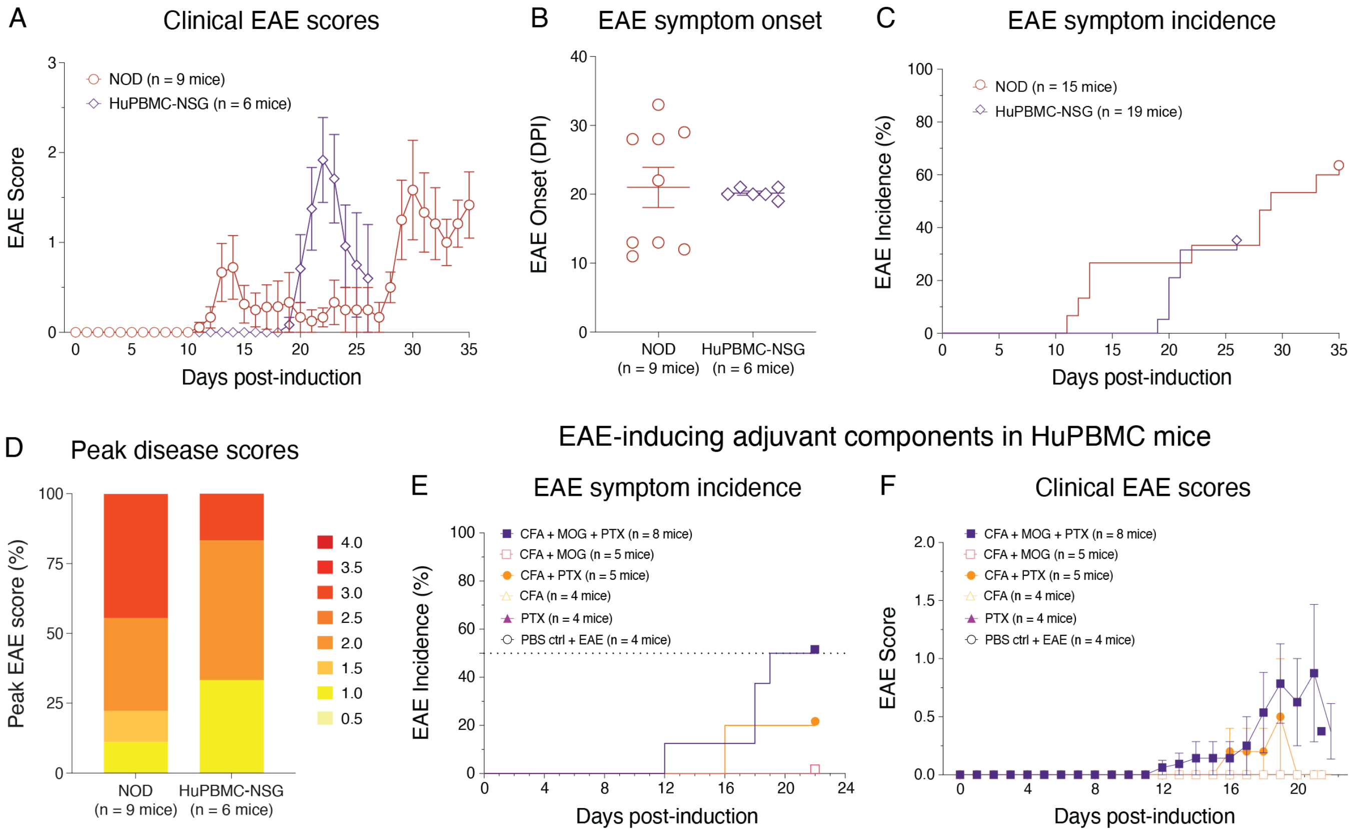

Figure S3.

Adjuvant effects and clinical EAE outcomes in HuPBMC mice compared to NOD mice. Figure shows comparative clinical outcomes following MOG_{35-55} peptide EAE immunization of wild-type Non-Obese Diabetic (NOD mice) and HuPBMC-NSG mice derived from an EBV^+ HD. (A) Clinical disease scores over time and (B) time to EAE symptom onset days post-induction (DPI) (n = 6 – 9 symptomatic mice/group). (C) EAE symptom incidence over time (n = 15 – 19 induced mice/ group) and (D) the distribution of peak EAE scores attained with each strain (n = 6 – 9 symptomatic mice/group). Figure also shows (E) clinical EAE symptom incidence and (F) average clinical disease scores over time for HuPBMC mice (n = 4 – 8 mice/group derived from the same EBV^+ HD, including both symptomatic and subclinical mice) immunized with individual or combined components of the EAE inducing adjuvant formulation with or without rhMOG/MOG_{35-55} antigen. In A, B, and F, data are shown as mean with SEM. In C, D, and E, data are shown as percentage of the group.

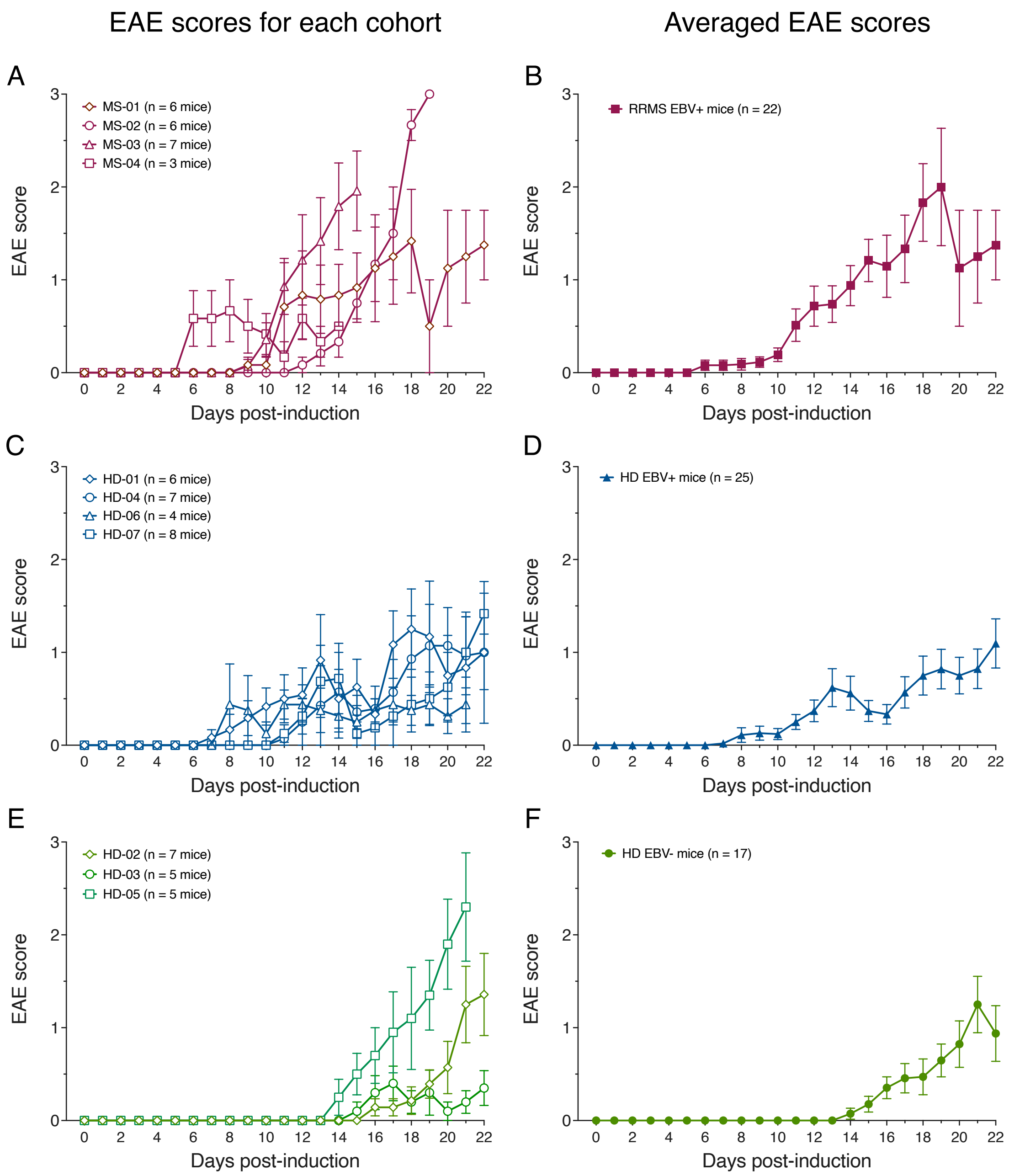

Figure S4.

Clinical EAE scores for each donor-derived HuPBMC cohort. Figure shows clinical disease curves following rhMOG/MOG_{35-55} EAE induction for symptomatic HuPBMC mice, grouped based on donor EBV serostatus and RRMS diagnosis. Figure shows (A) separate clinical EAE scores over time for each of the four RRMS EBV^+ donor-derived cohorts (n = 3 – 7 mice/group from each donor) and corresponding (B) averaged clinical scores for all RRMS EBV^+ mice (n = 22 symptomatic mice derived from n = 4 donors total). (C) Separate

clinical EAE scores over time for each of the four EBV^+ HD-derived cohorts (n = 4 – 8 mice/group from each donor) and corresponding (D) averaged clinical scores for all EBV^+ HD mice (n = 25 symptomatic mice derived from n = 4 donors total). (E) Separate clinical EAE scores over time for each of the three EBV^- HD-derived cohorts (n = 5 – 7 mice/group from each donor) and corresponding (D) averaged clinical scores for all EBV^- HD mice (n = 17 symptomatic mice derived from n = 3 donors total). All data are shown as mean with SEM.

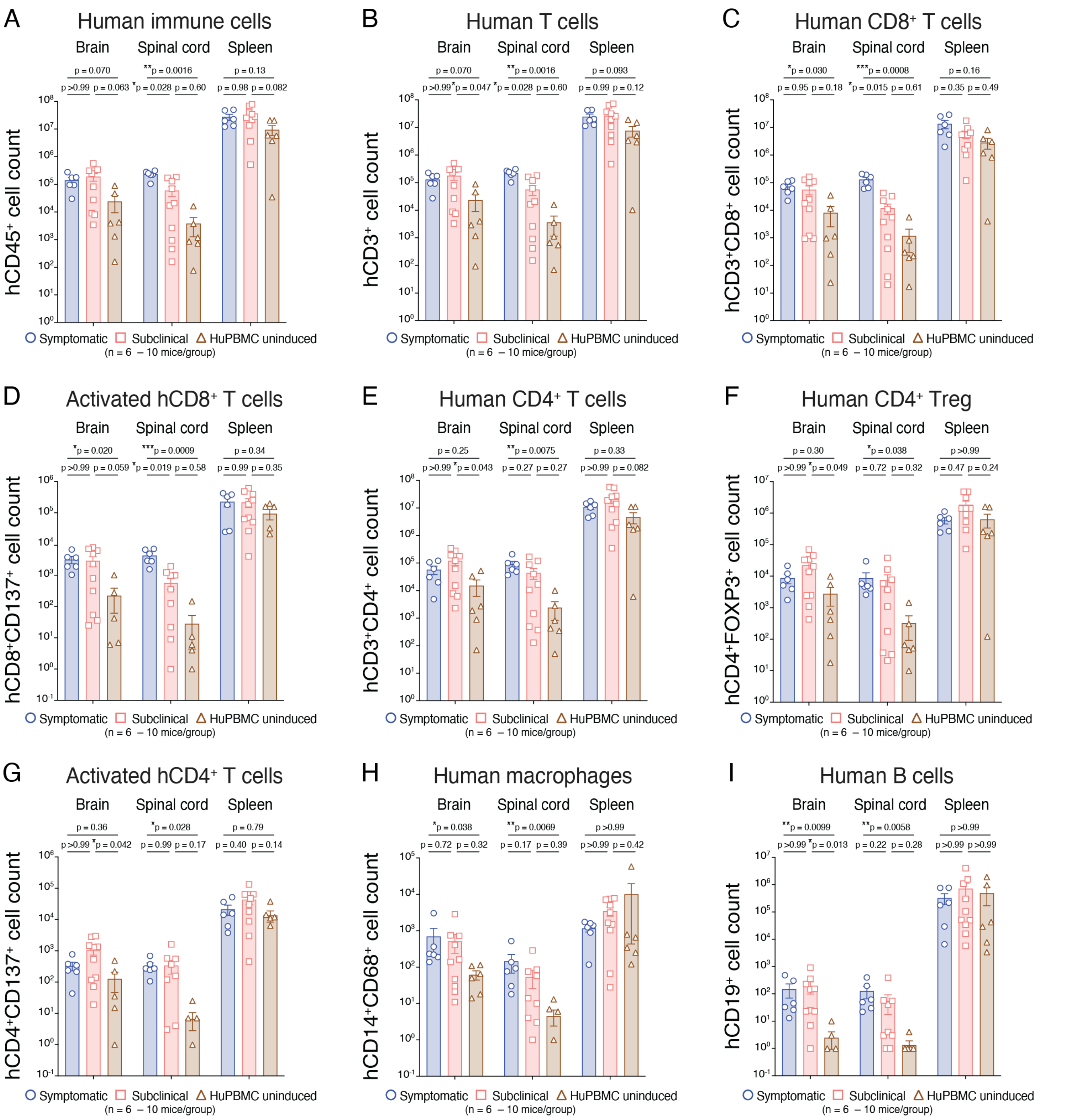

Figure S5.

Spinal cord infiltrating human immune cell counts define clinical symptom presentation in HuPBMC EAE mice. Total numbers of (A) $hCD45^+$ immune cells, (B) $hCD3^+$ T cells, (C) $hCD3^+CD8^+$ T cells, (D) activated $hCD3^+CD8^+CD137^+$ T cells, (E) $hCD3^+CD4^+$ T cells, (F) $hCD3^+CD4^+FOXP3^+$ regulatory T cells, (G)

activated $hCD3^+CD4^+CD137^+$ T cells, (H) $hCD14^+CD68^+$ macrophages and (I) $hCD19^+$ B cells, in the CNS and spleens of uninduced HuPBMC mice and of EAE-induced HuPBMC mice that either developed symptoms or remained subclinical. Perfused tissues were collected days 14 and 24 post-induction of recipient cohorts derived from two unrelated EBV^+ HD PBMCs, and data were combined for analysis. Data are shown as mean with SEM (n = 6 – 10 mice/group) and were analyzed by Brown-Forsythe and Welch ANOVA with Dunnett's T3 multiple comparisons test or by Kruskal-Wallis with Dunn's multiple comparisons test.

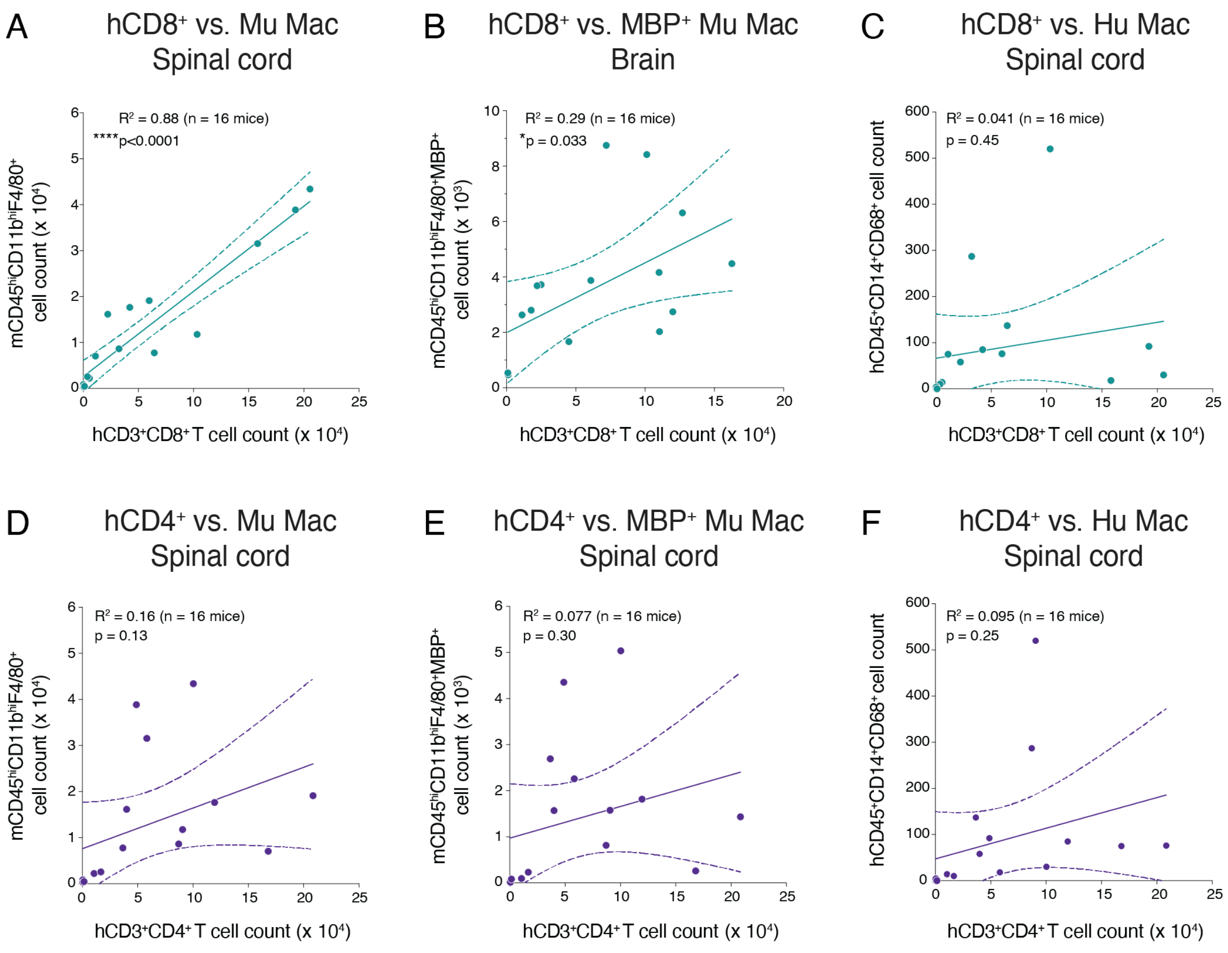

Figure S6.

Relationship between CNS infiltrating human T cell populations and murine or human macrophages in HuPBMC EAE mice. Top row shows correlations between the total number of hCD3$^+$CD8$^+$ T cells and (A) murine macrophages (Mac; mCD45^{hi}CD11b^{hi}F4/80$^+$) in the spinal cord, (B) murine macrophages in the brain containing intracellular myelin basic protein (MBP), and (C) human macrophages (hCD45$^+$CD14$^+$CD68$^+$) in the spinal cord. Bottom row shows correlations between the total number of hCD3$^+$CD4$^+$ T cells in the spinal cord and (D) murine macrophages, (E) murine macrophages containing intracellular MBP, and (F) human macrophages. Perfused tissues were collected days 14 and 24 post-EAE induction of recipient cohorts derived from two unrelated EBV$^+$ HD PBMCs, and data were combined for analysis. Data were analyzed by simple linear regression (n = 16 mice). Goodness of fit is indicated by R^2 value, and the 95% confidence interval by dashed lines.

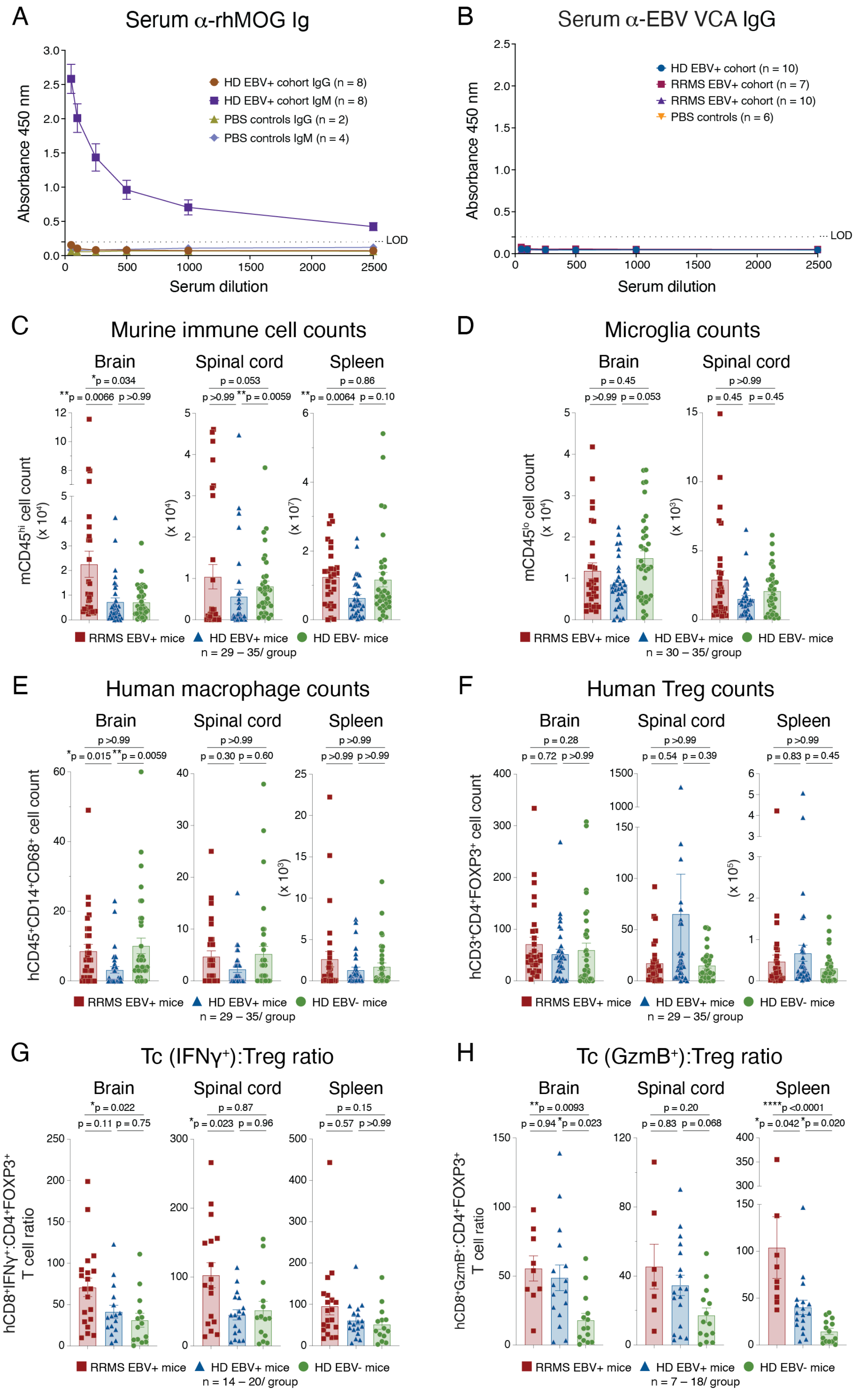

A
Serum α-rhMOG Ig
B
Serum α-EBV VCA IgG
C
Murine immune cell counts
D
Microglia counts
E
Human macrophage counts
F
Human Treg counts
G
Tc (IFNγ+):Treg ratio
H
Tc (GzmB+):Treg ratio

Figure S7.

Human antibody generation and immune cell infiltration in HuPBMC EAE mice. Figure shows (A) the deficiency in human Ig class-switching from IgM to IgG in response to the rhMOG inducing antigen in HuPBMC-NSG EAE mice (n = 8 mice derived from one HD EBV^+ donor) and (B) the absence of human IgG specific to EBV viral capsid antigen (VCA) in HuPBMC EAE mice derived from three unrelated EBV^+ donors (n = 6 – 10 mice/group derived from one donor each). In A and B, serum samples were collected days 15 – 25 post-EAE induction of HuPBMC mice (average 5 – 8 days post-symptom onset) or from PBS-injected NSG controls (n = 2 – 6 mice/group). Data are shown as mean with SEM. As most data points fell below the limit of detection (LOD, dashed line), data were not assessed statistically. Figure also shows (C) murine $CD45^{hi}$ immune cell counts, (D) murine $CD45^{lo}$ cell counts, (E) human macrophage ($hCD14^+CD68^+$) cell counts, (F) human regulatory T cell ($hCD4^+FOXP3^+$) counts, (G) the ratio of $hCD8^+IFN\gamma^+$ T cells to $hCD4^+FOXP3^+$ T cells, and (H) the ratio of $hCD8^+GzmB^+$ T cells to $hCD4^+FOXP3^+$ T cells in the CNS and spleens of HuPBMC EAE mice. In C – H, perfused organs were collected days 14 – 27 post-EAE induction (average 5 – 10 days post-symptom onset). For total immune cell quantification, n = 29 – 35 mice/group derived from 2 – 3 donors/group. For cytokine analysis, isolated cells were stimulated with PMA and ionomycin from n = 7 – 20 mice/group derived from 1 – 2 donors/group. Data are shown as mean with SEM and were analyzed by Brown-Forsythe and Welch ANOVA with Dunnett's T3 multiple comparisons test or by Kruskal-Wallis with Dunn's multiple comparisons test.

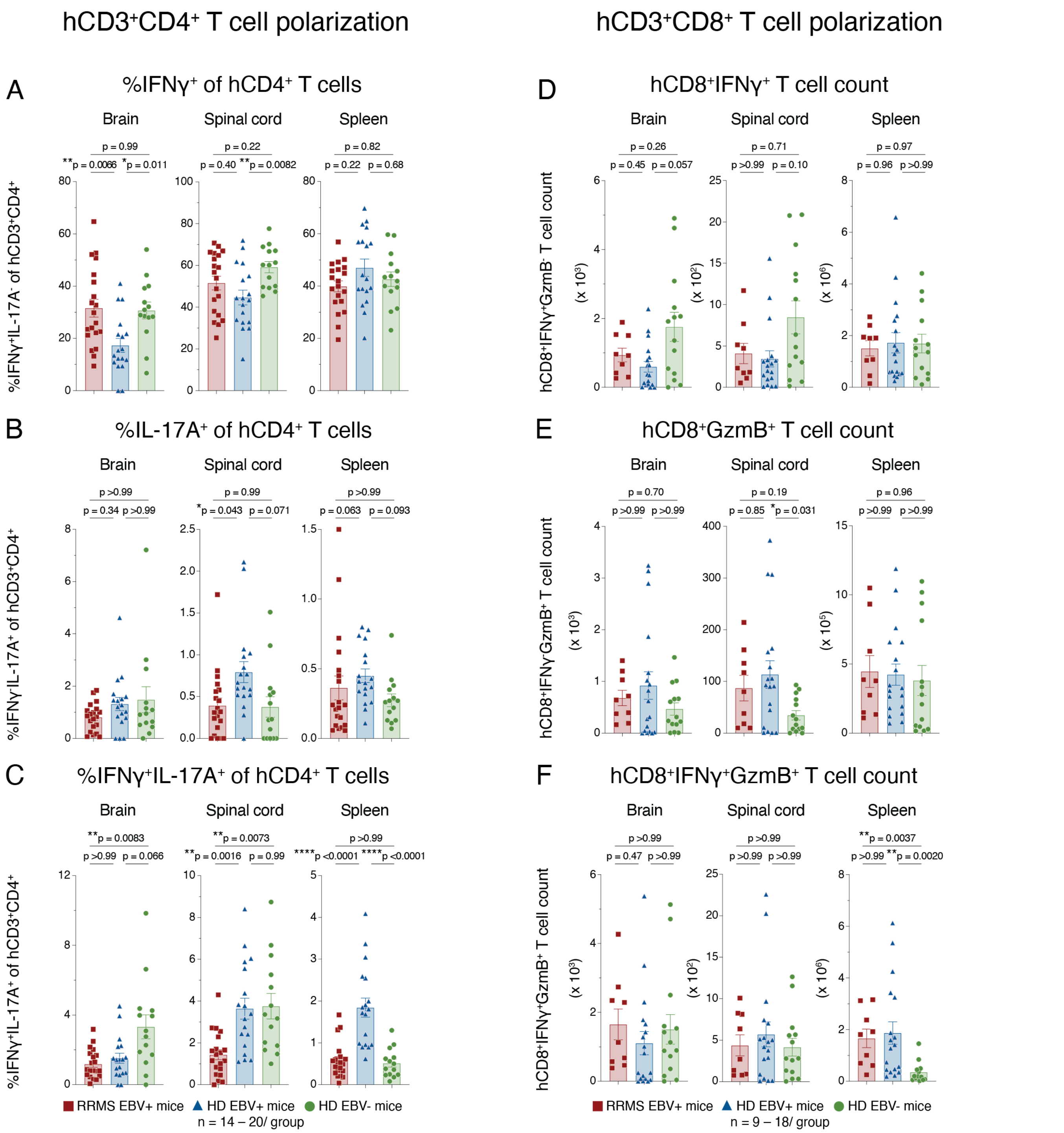

Figure S8.

Donor EBV and RRMS status promote effector T cell expansion in the HuPBMC EAE model, continued. Figure shows brain and spinal cord infiltration and spleen reconstitution in recipient HuPBMC EAE

mice at endpoint, grouped by PBMC donor EBV serostatus and RRMS diagnosis. (A) %IFNγ^+(IL-17A$^-$), (B) %IL-17A$^+$(IFNγ^-), and (C) %IFNγ^+IL-17A$^+$ of hCD3$^+$CD4$^+$ T cells, as well as total (D) IFNγ^+(GzmB$^-$), (E) GzmB$^+$(IFNγ^-), (F) IFNγ^+GzmB$^+$ hCD8$^+$ T cell counts in each tissue. Perfused organs were collected days 14 – 27 post-EAE induction (average 5 – 10 days post-symptom onset). Isolated immune cells were stimulated with PMA and ionomycin for cytokine detection (n = 9 – 20 mice/group derived from 1 – 2 donors/group). All plotted data are shown as mean with SEM and were analyzed by Brown-Forsythe and Welch ANOVA with Dunnett's T3 multiple comparisons test or by Kruskal-Wallis with Dunn's multiple comparisons test.

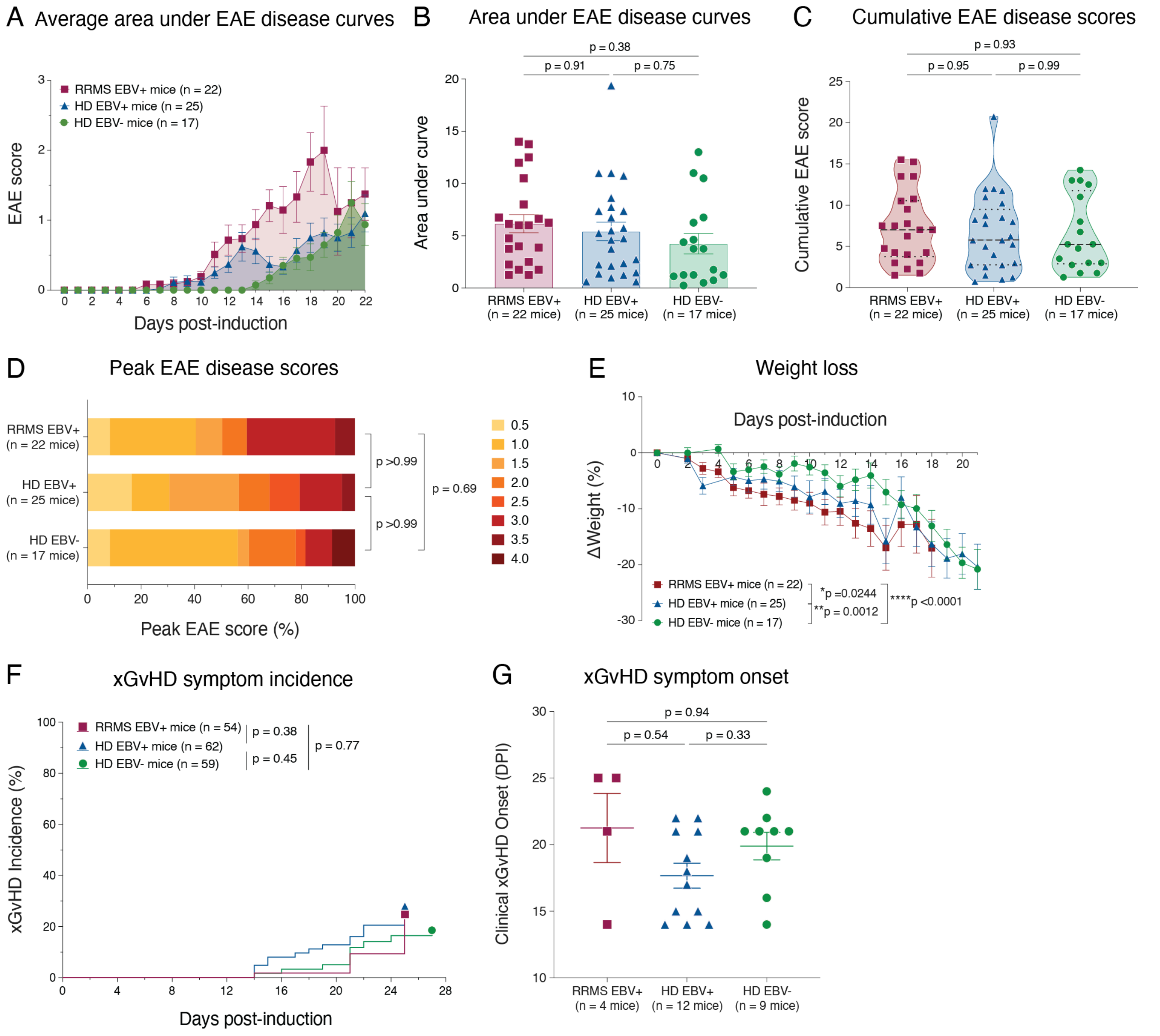

Figure S9.

Clinical EAE and xGvHD outcomes in HuPBMC EAE mice. Figure shows the (A) average and (B) individual areas under the clinical EAE disease curves, (C) the distribution of cumulative EAE disease scores, (D) the proportion of EAE scores attained at the peak of clinical disease, and (E) weight loss over time, among symptomatic EAE mice group by donor EBV and RRMS status (n = 17 – 25 mice/group derived from 3 – 4 donors/group). Figure also shows the (F) incidence of symptoms of xenogeneic graft-versus-host disease (xGvHD) over time (n = 54 – 62 mice/group derived from 3 – 4 donors/group) and (G) the time to xGvHD symptom onset (n

= 4 – 12 symptomatic xGvHD mice/group derived from 3 – 4 donors/group among the HuPBMC EAE recipient groups. Recipient group average EAE symptom duration to endpoint was: RRMS EBV^+ mice 6.0 ± 1.6 days, HD EBV^+ mice 6.9 ± 2.4 days and HD EBV^- mice 7.5 ± 1.4 days. In A, B, E, and G, data are shown as mean with SEM. In C, distribution of individual data is shown with median and quartiles (dashed lines). In B, C, and G, data were analyzed by Brown-Forsythe and Welch ANOVA with Dunnett's T3 multiple comparisons test. In D, data are represented as proportions of all EAE scores attained in each group, and individual peak EAE scores per mouse were plotted and analyzed by Kruskal-Wallis with Dunn's multiple comparisons test. In E, curves were analyzed by ordinary two-way ANOVA. In F, data are shown as percentage of the group and curves were analyzed by Log-rank (Mantel-Cox) test.

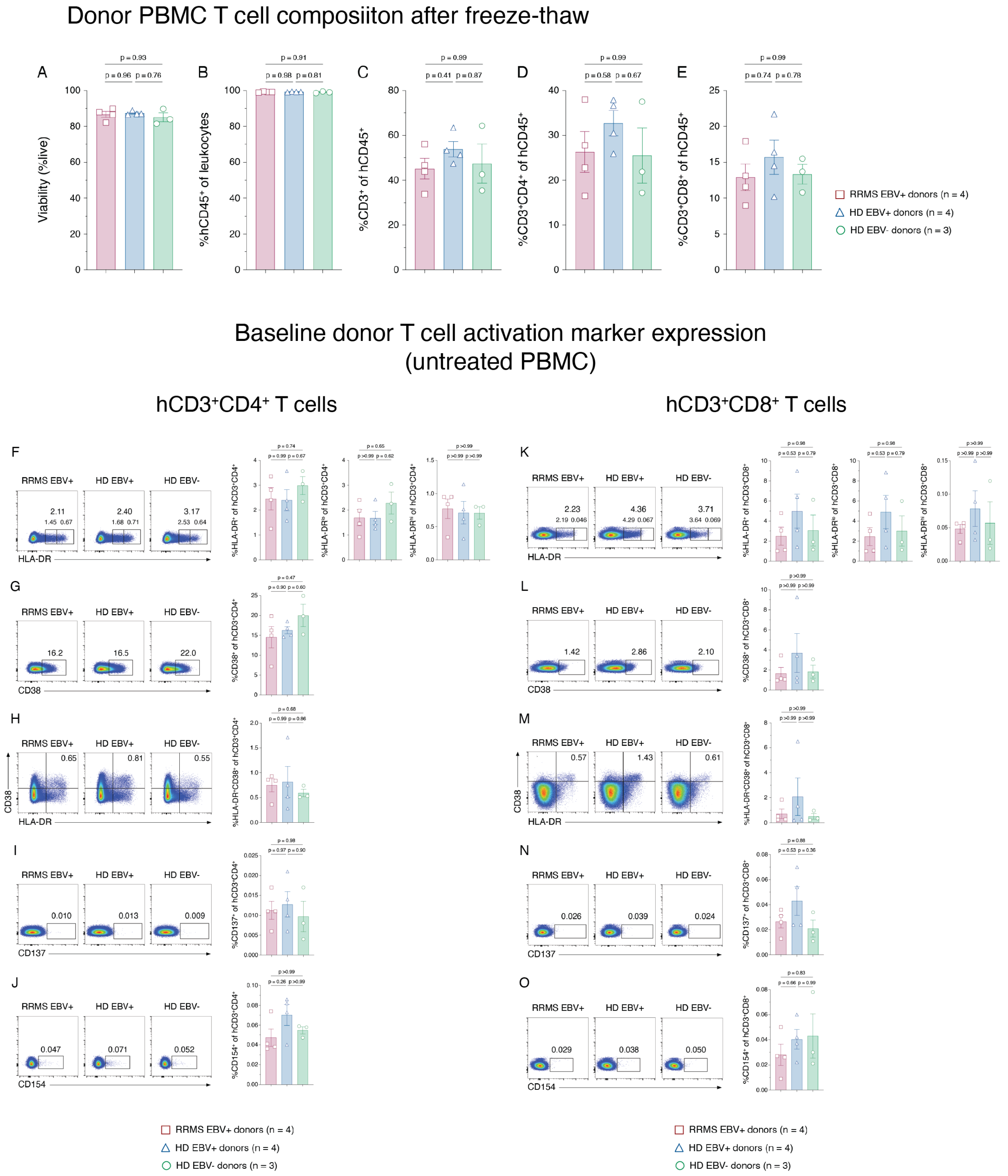

Donor PBMC T cell composiiton after freeze-thaw
A
Viability (%live)
B
%hCD45⁺ of leukocytes
C
%CD3⁺ of hCD45⁺
D
%CD3⁺CD4⁺ of hCD45⁺
E
%CD3⁺CD8⁺ of hCD45⁺
RRMS EBV+ donors (n = 4)
HD EBV+ donors (n = 4)
HD EBV- donors (n = 3)
Baseline donor T cell activation marker expression
(untreated PBMC)
hCD3⁺CD4⁺ T cells
hCD3⁺CD8⁺ T cells
F
G
H
I
J
K
L
M
N
O
RRMS EBV+
HD EBV+
HD EBV-
HLA-DR
CD38
CD137
CD154
RRMS EBV+ donors (n = 4)
HD EBV+ donors (n = 4)
HD EBV- donors (n = 3)
RRMS EBV+ donors (n = 4)
HD EBV+ donors (n = 4)
HD EBV- donors (n = 3)

Figure S10.

Donor PBMC composition and baseline T cell activation marker expression. Following donor PBMC preservation in liquid nitrogen, group differences in PBMC composition were assessed by (A) the viability of the cells (proportion alive) after recovery, (B) the proportion of $hCD45^+$ cells among all leukocytes, (C) the proportion of $hCD3^+$ T cells among $hCD45^+$ cells, (D) the proportion of $hCD3^+CD4^+$ T cells among $hCD45^+$ cells, and (E) the proportion of $hCD3^+CD8^+$ T cells among $hCD45^+$ cells. Baseline donor T cell activation was assessed by the proportion of untreated $hCD3^+CD4^+$ (left) and $hCD3^+CD8^+$ (right) T cells expressing the markers (F, K) HLA-DR ($^+$, ^{lo}, and ^{hi}), (G, L) CD38, (H, M) both HLA-DR and CD38, (I, N) CD137, and (J, O) CD154. All plotted data are shown as mean with SEM (n = 3 – 4 blood donors/group) and were analyzed by Brown-Forsythe and Welch ANOVA with Dunnett's T3 multiple comparisons test or by Kruskal-Wallis with Dunn's multiple comparisons test. Concatenated flow plots illustrate the sum proportion of marker positive cells for all donors in each group.

Basline donor T cell polarization

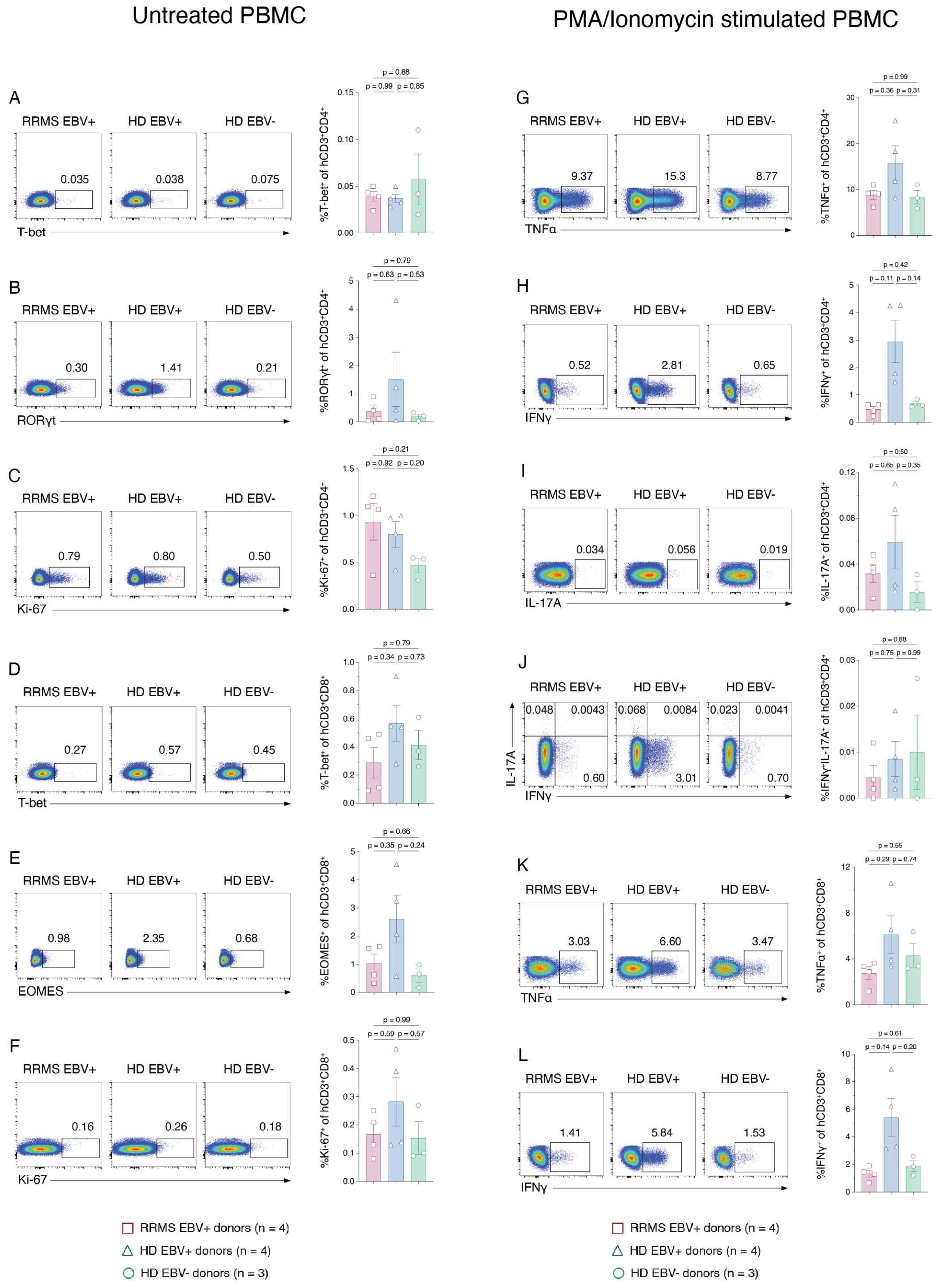

Figure S11.

Baseline donor T cell polarization marker expression. Following donor PBMC preservation in liquid nitrogen, baseline donor T cell polarization was assessed by measuring the proportion of untreated (left) and PMA-Ionomycin stimulated (right) cells expressing the markers (A) T-bet, (B) RORγt, and (C) Ki-67 on $hCD3^{+}CD4^{+}$ T cells; (D) T-bet, (E) EOMES, and (C) Ki-67 on $hCD3^{+}CD8^{+}$ T cells; (G) TNFα, (H) IFNγ, (I) IL-17A and (J) both IFNγ and IL-17A on $hCD3^{+}CD4^{+}$ T cells; and (K) TNFα and (L) IFNγ on $hCD3^{+}CD8^{+}$ T cells. All plotted data are shown as mean with SEM (n = 3 – 4 blood donors/group) and were analyzed by Brown-Forsythe and Welch ANOVA with Dunnett's T3 multiple comparisons test or by Kruskal-Wallis with Dunn's multiple comparisons test. Concatenated flow plots illustrate the sum proportion of marker positive cells for all donors in each group.

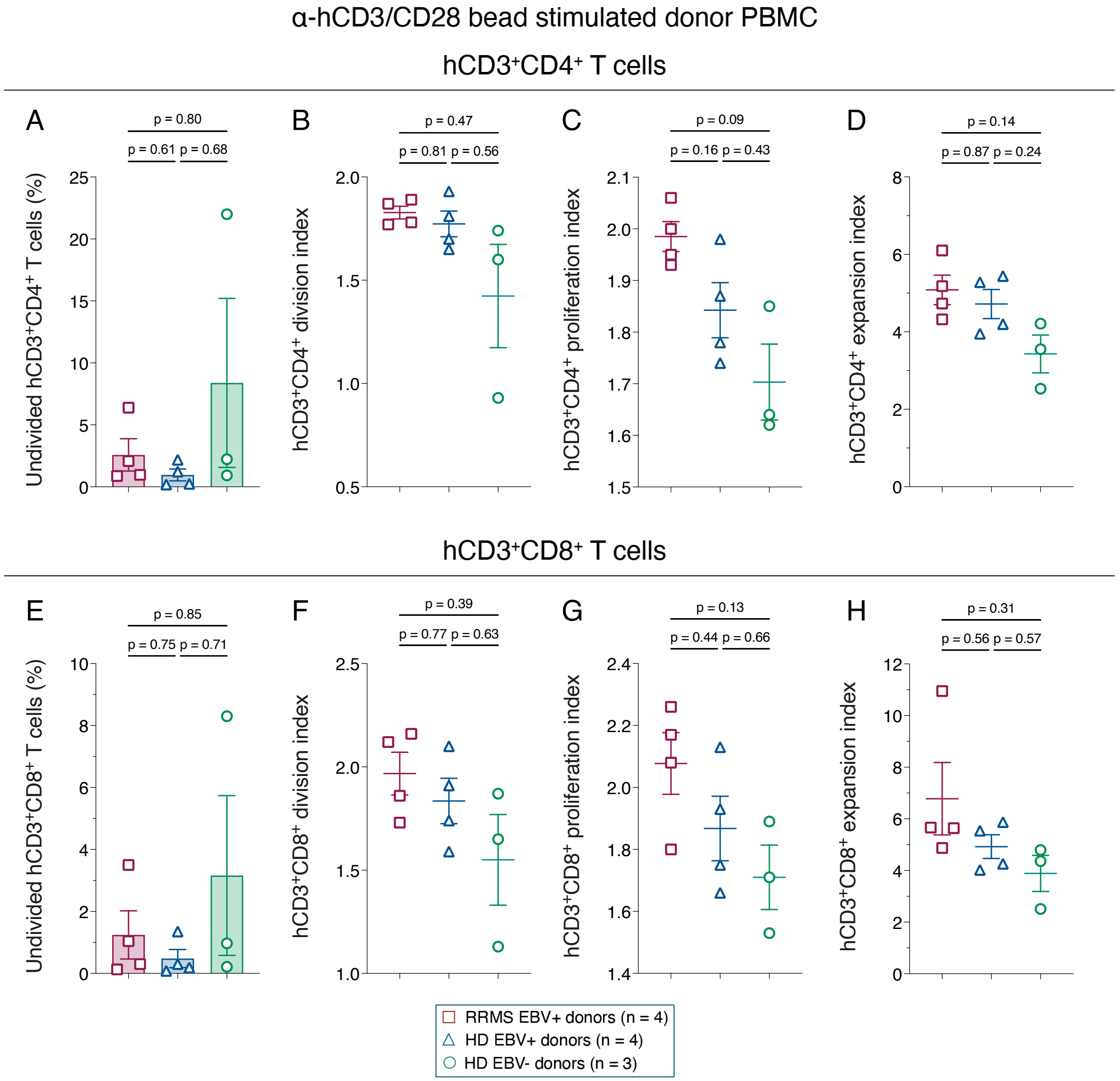

Figure S12.

CFSE-modelled proliferation of donor PBMCs stimulated with anti-CD3/CD28 coated beads. Previously frozen, whole PBMC samples from EBV^+ RRMS, EBV^+ HD, and EBV^- HD blood donors were incubated with anti-CD3/CD28 coated beads for 96 hours to stimulate T cells in the absence of a specific antigen. Figure shows (A) the proportion of undivided $hCD3^+CD4^+$ T cells and the (B) division, (C) proliferation, and (D) expansion indices for $hCD3^+CD4^+$ T cells based on CFSE staining. Figure also shows (E) the proportion of undivided $hCD3^+CD8^+$ T cells and the (F) division, (G) proliferation, and (H) expansion indices for $hCD3^+CD8^+$ T

cells based on CFSE staining. The colored symbol legend is applicable to all comparisons (n = 3 – 4 blood donors/group). All plotted data are shown as mean with SEM and were analyzed by Brown-Forsythe and Welch ANOVA with Dunnett's T3 multiple comparisons test or by Kruskal-Wallis with Dunn's multiple comparisons test.

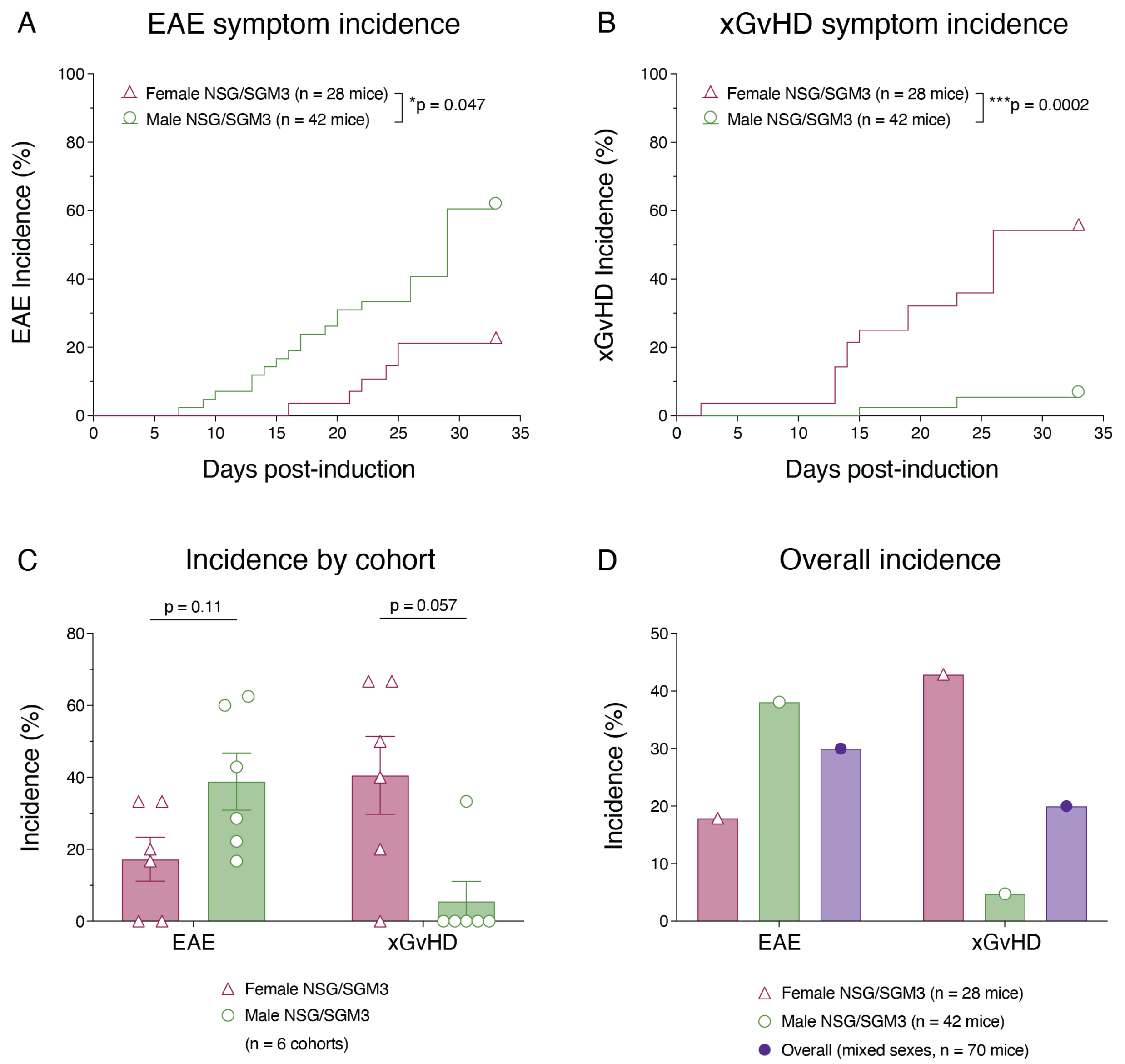

Figure S13.

Effect of recipient NSG sex on EAE and xGvHD symptom incidence in the HuPBMC model. Figure shows clinical EAE (A) and xenogeneic (x)GvHD (B) symptom incidence over time as a proportion of all female recipient (n = 28) and male recipient (n = 42) NSG/SGM3 mice post-rhMOG and/or $MOG_{35\text{-}55}$ EAE induction. Recipient NSG and NSG-SGM3 mice were grouped together (treated interchangeably) and are denoted as NSG/SGM3. Data shown are combined from six separate HuPBMC cohorts engrafted with unrelated healthy female donor PBMCs (n = 4 HDs) and were analyzed by Log-rank (Mantel-Cox) test. (C) Recipient sex-based incidence of EAE and xGvHD symptoms for each of the six cohorts, shown as mean with SEM and analyzed by Mann-

Whitney test, as well as the (D) overall recipient sex-based incidence of EAE and xGvHD symptoms for all HuPBMC mice combined.

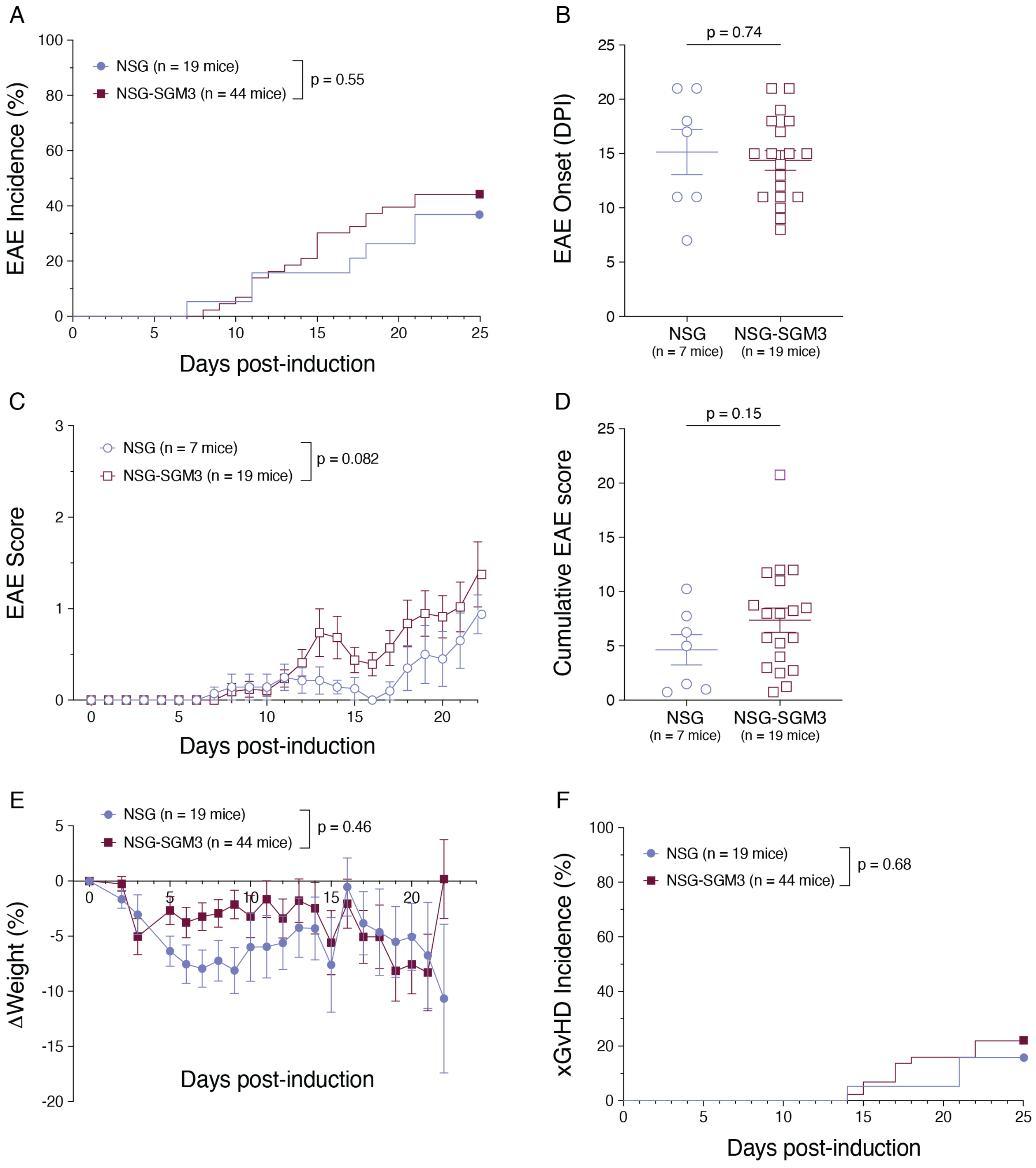

Figure S14.

Clinical EAE outcomes for PBMC engrafted NSG and NSG-SGM3 mice. Figure shows (A) incidence of EAE symptoms over time among all mixed rhMOG/MOG$_{35\text{-}55}$ immunized PBMC engrafted male NSG mice (n = 19) and NSG-SGM3 mice (n = 44) derived from healthy female donors (n = 4 HDs). Data are shown as percentage

of the group and curves were analyzed by Log-rank (Mantel-Cox) test. Among the HuPBMC NSG (n = 7) and NSG-SGM3 mice (n = 19) that developed symptoms of EAE, figure shows (B) day of EAE symptom onset post-induction (DPI), (C) clinical EAE scores over time, and (D) cumulative EAE scores for each strain group. Data are shown as mean with SEM. Data in B and D were analyzed by Welch's unpaired t test, data in C were analyzed using a mixed-effects two-way model. Among all EAE induced HuPBMC NSG (n = 19) and NSG-SGM3 mice (n = 44), figure shows (E) weight loss and (F) symptoms of xGvHD over time for each strain group. Data in E are shown as mean with SEM and were analyzed using a mixed-effects two-way model. Data in F are shown as percentage of the group and curves were analyzed by Log-rank (Mantel-Cox) test.

SUPPLEMENTARY TABLES

Table S1.

Antibodies for extracellular targets.

Target antigen	Clone	Species reactivity	Application	Source	Identifier
CD3	OKT3	Human	Flow cytometry	Thermo Fisher	56-0037-42
CD4	RPA-T4	Human	Flow cytometry	BD Biosciences	555347
CD4	RPA-T4	Human	Flow cytometry	Thermo Fisher	47-0049-42
CD8	SP16	Human	IHC-F	Thermo Fisher	MA5-14548
CD8	RPA-T8	Human	Flow cytometry	Thermo Fisher	11-0088-42
CD8	RPA-T8	Human	Flow cytometry	BD Biosciences	563821
CD11b	M1/70	Mouse	Flow cytometry	Thermo Fisher	17-0112-82
CD11c	N418	Mouse	Flow cytometry	Thermo Fisher	11-0114-85
CD14	63D3	Human	Flow cytometry	BioLegend	367142
CD14	61D3	Human	Flow cytometry	Thermo Fisher	11-0149-42
CD19	HIB19	Human	Flow cytometry	Thermo Fisher	47-0199-42
CD20	2H7	Human	Flow cytometry	BioLegend	302328
CD21	HB5	Human	Flow cytometry	Thermo Fisher	12-0219-42
CD25	CD25-4E3	Human	Flow cytometry	Thermo Fisher	11-0257-42
CD25	BC96	Human	Flow cytometry	Thermo Fisher	45-0259-42
CD27	LG.3A10	Human	Flow cytometry	BioLegend	124216
CD38	HIT2	Human	Flow cytometry	BioLegend	303529
CD40	3/23	Mouse	Flow cytometry	Thermo Fisher	MA5-17855
CD45	HI30	Human	Flow cytometry	Thermo Fisher	MHCD4517
CD45	HI30	Human	Flow cytometry	BD Biosciences	557748
CD45	30-F11	Mouse	Flow cytometry	Thermo Fisher	45-0451-82
CD45	30-F11	Mouse	Flow cytometry	Thermo Fisher	48-0451-82
CD56	TULY56	Human	Flow cytometry	Thermo Fisher	17-0566-42
CD137	4B4-1	Human	Flow cytometry	BioLegend	309818
CD154 (40L)	24-31	Human	Flow cytometry	BioLegend	310823
F4/80	BM8	Mouse	Flow cytometry	Thermo Fisher	47-4801-82
Fc receptor (CD16/CD32)	2.4G2	Mouse	Flow cytometry	BD Biosciences	553142
Fc receptor	Fc1.3216	Human	Flow cytometry	BD Biosciences	564220

HLA-DR	L243	Human	Flow cytometry	Thermo Fisher	17-9952-42
Iba-1	Polyclonal	Mouse	IHC-F	Thermo Fisher	PA5-18039
IgG (H+L)	Polyclonal	Goat	IHC-F	Jackson ImmunoResearch	205-585-108
IgG (γ chain)	Polyclonal	Human	ELISA	Thermo Fisher	62-8420
IgG (H+L)	Polyclonal	Rabbit	IHC-F	Thermo Fisher	A10043
IgG2b	m2b-25G4	Mouse	Flow cytometry	Thermo Fisher	11-4220-82
IgM (μ chain)	Polyclonal	Human	ELISA	Thermo Fisher	A18841

Table S2.

Antibodies for intracellular targets.

Target antigen	Clone	Species reactivity	Application	Source	Identifier
CD68	Y1/82A	Human	Flow cytometry	BioLegend	333825
EOMES	WD1928	Human	Flow cytometry	Thermo Fisher	11-4877-41
FOXP3	236A/E7	Human	Flow cytometry	Thermo Fisher	17-4777-42
Granzyme B	QA16A02	Human	Flow cytometry	BioLegend	372214
IFNγ	4S.B3	Human	Flow cytometry	Thermo Fisher	48-7319-42
IL-17A	eBio64DEC17	Human	Flow cytometry	Thermo Fisher	25-7179-42
Ki-67	Ki-67	Human	Flow cytometry	BioLegend	350516
MBP	MBP101	Mouse	Flow cytometry	Abcam	ab62631
RORγt	AFKJS-9	Human	Flow cytometry	Thermo Fisher	12-6988-80
T-bet	4B10	Human	Flow cytometry	Thermo Fisher	45-5825-82
TNFα	MAb11	Human	Flow cytometry	Thermo Fisher	17-7349-82

Table S3.

Antigen sequences for endogenous antibody detection by ELISA.

Antigen	Sequence
EBV VCA p18 (116)	ASAGTGALASSAPSTAVAQSATPSVSSSISSLRAATSGATAAAAVDTGS GGGGQPHDTAPRGARKKQ
EBV EBNA-1 (116)	Epitope 1: RSPSSQSSSSGSPPRRPPPGRRPFFHPVG
	Epitope 2: DYFEYHQEGGPDGEPDVPPGAIEQGPADDPGEGPSTGPRG
rhMOG$_{1\text{-}120}$ (114)	MASLSRPSLPSCLCSFLLLLLLQVSSSYAGQFRVIGPRHPIRALVGDEVE LPCRISPGKNATGMEVGWYRPPFSRVVHLYRNGKDQDGDQAPEYRG RTELLKDAIGEGKVTLRIRNVRFS
CMV (117)	Epitope 1: CETDDLDEEDTSIYLSPPPVPPVQVVAKRLPRPDTPRT
	Epitope 2: KSGTGPQPGSAGMGGAKTPSDAVQNILQKIEKIKNTEE

Table S4.

DNA sequences for EBV *BALF5* qPCR assay.

Component	Sequence (5’ to 3’)
Primer P1 (50)	CGGAAGCCCTCTGGACTTC
Primer P2 (50)	CCCTGTTTATCCGATGGAATG
Probe P3 (50)	/5HEX/TGTACACGC/ZEN/ACGAGAAATGCGCC/3IABkFQ/
BALF5 gene standard (gBlock)	ACC GAG ACC CGG CAG GGG GTC CTG CGG TCG AAG GTG CTG GCC TTG AGG GCG CTG AGG ACT GCA AAC TCC ACG TCC AGA CCC TGA GGC GCG CTG GCG TAG AAG TAG GCC TGC TGC CCA AAC ACG TTC ACA CAC ACG CTG GCC CCA TCG GCC TTG CGC CGG CCC AGT AGC TTG ATG ACG ATG CCA CAT GGC ACC ACA TAC CCC TGT TTA TCC GAT GGA ATG ACG GCG CAT TTC TCG TGC GTG TAC ACC GTC TCG AGT ATG TCG TAG ACA TGG AAG TCC AGA GGG CTT CCG TGG GTG TCT GCC TCC GGC CTT GCC GTG CCC TCT TGG GCA CGC TGG CGC CAC CAC ATG CCC TTT CCA TCC TCG TCA CCC CCC ACC ACC GTC AGG GAG TCT TGG TAG AAG CAC AGG GGG GGC TGA GGC CCC CGC ACA TCC ACC ACC CCT GCG GCG CCT GGT GTC TGG AAA CAC TTG GGA ATG AGAC GCA GGT ACT CCT TGT CAG GCT TTT TC

ABBREVIATIONS

CMV	Cytomegalovirus
CNS	Central nervous system
DC	Dendritic cell
DPI	Day post-induction
EAE	Experimental autoimmune encephalomyelitis
EBNA-1	Epstein-Barr nuclear antigen 1
EBV	Epstein-Barr virus
EDSS	Expanded disability status scale
GzmB	Granzyme B
HD	Healthy donor
HLA	Human leukocyte antigen
HuPBMC	Human peripheral blood mononuclear cell mouse model
IM	Infectious mononucleosis
MBP	Myelin basic protein
MHC	Major histocompatibility complex
(rh)MOG	(Recombinant human) Myelin oligodendrocyte glycoprotein
(RR)MS	(Relapsing-remitting) Multiple sclerosis
NOD	Non-Obese Diabetic (mouse strain)
NSG	NOD/SCID-IL-2Rγc $^{-/-}$ (NOD.Cg-*Prkdc*^{scid} *Il2rg*^{tm1Wjl}/SzJ mouse strain)
PBMC	Peripheral blood mononuclear cell
SD	Standard deviation
SEM	Standard error of the mean
TCR	T cell receptor
Treg	Regulatory $CD3^+CD4^+$ T cell
VCA	Viral capsid antigen
xGvHD	Xenogeneic graft-versus-host disease
γHV68	Murine gammaherpesvirus-68
